# Highlighting sarcopenia management in cancer treatments: evidence from umbrella meta-analysis

**DOI:** 10.3389/fnut.2026.1831634

**Published:** 2026-07-16

**Authors:** Tongfa Yan, Jiahao Cui, Xiankai Chen, Zhengpan Wei, Zhiqian Zhao, Bin Yan, Xue Pan, Peiyu Wang, Xiangnan Li

**Affiliations:** 1 Department of Thoracic Surgery, The First Affiliated Hospital of Zhengzhou University, Zhengzhou, Henan, China; 2 Department of Statistics, Columbia University, New York, NY, United States; 3 Department of Thoracic Surgical Oncology, National Cancer Center/Cancer Hospital, Beijing, China; 4 School of Nursing and Health, Zhengzhou University, Zhengzhou, Henan, China

**Keywords:** cancer, meta-analysis, sarcopenia, systematic review, umbrella review

## Abstract

**Introduction:**

The role of sarcopenia in cancer treatment remains to be fully documented. This umbrella meta-analysis aimed to investigate the impact of sarcopenia on therapeutic outcomes across common malignancies with high incidence or mortality.

**Methods:**

We conducted an umbrella review of existing meta-analyses based on cohort studies. PubMed, Web of Science, and Embase were systematically searched from inception to October 2025. The primary endpoints were survival outcomes after surgical or first-line treatments. Supplementary meta-analyses were performed for outcomes with insufficient existing evidence. The systematic review protocol was registered in PROSPERO (CRD42022383726).

**Results:**

A total of 59 meta-analyses were included, comprising 49 from the literature search and 10 newly conducted in this study, covering 12 cancer types. In patients undergoing surgery, sarcopenia was significantly associated with shorter overall survival (HR = 1.59, 95% CI: 1.37–1.88) and disease-free survival (HR = 1.64, 95% CI: 1.39–1.93), as well as increased risks of any postoperative complications (OR = 1.48, 95% CI: 1.21–1.81) and major complications (OR = 1.51, 95% CI: 1.30–1.76). In patients receiving first-line non-surgical therapy, sarcopenia remained an independent risk factor for poorer overall survival (HR = 1.49, 95% CI: 1.39–1.61) and progression-free survival (HR = 1.39, 95% CI: 1.17–1.66). The methodological quality of included meta-analyses was generally high, while the GRADE certainty of evidence for most outcomes was rated as low or very low. Sarcopenia was also associated with inferior survival after cancer immunotherapy.

**Discussion:**

Sarcopenia is consistently associated with adverse therapeutic outcomes across multiple cancers and treatment modalities. It warrants attention in clinical management and represents a promising target for improving cancer therapeutic outcomes.

**Systematic review registration:**

https://www.crd.york.ac.uk/prospero/, identifier CRD42022383726.

## 1 Introduction

Cancer has emerged as a leading global public health challenge. According to the 2022 global cancer statistics, annual incidence is projected to exceed 35 million by 2025 (1). Cancer accounts for three in ten premature deaths from non-communicable diseases (30.3%) among individuals aged 30–69 years, ranking among the top three causes of death in this age group in 177 out of 183 countries (2). Furthermore, the economic burden of cancer is substantial, with the global economic cost estimated to reach US$25.2 trillion between 2020 and 2050 (3). Therefore, the prevention and management of this disease are of significant public health importance.

Advances in diagnosis and treatment have improved patient survival; however, the catabolic state induced by the tumor and its treatment—characterized by chronic inflammation, reduced intake, and metabolic alterations—has heightened the prominence of cancer-associated malnutrition and deteriorating body composition (4–7). Sarcopenia, defined as the progressive loss of skeletal muscle mass and function, is already present at diagnosis in 25%–60% of cancer patients, with particularly high prevalence observed in those with esophageal or urothelial cancers (8). Imaging techniques, particularly computed tomography (CT) and magnetic resonance imaging (MRI), represent the most objective and widely used tools for identifying sarcopenia in clinical practice and research. Radiologically assessed sarcopenia, based on quantitative measurements of skeletal muscle mass, has been increasingly recognized as a reliable prognostic biomarker across various malignancies, including lymphoma and other solid tumors. This non-invasive radiological approach enables standardized evaluation of muscle status, providing critical information for risk stratification and clinical decision-making in oncology (9). A growing body of research has explored the association between sarcopenia and cancer outcomes, leading to several systematic reviews and meta-analyses examining overall survival (OS), progression-free survival (PFS), and other endpoints (10, 11). While these findings carry potential implications for cancer prognosis, the strength, precision, and risk of bias in these associations require further clarification.

Recent studies have demonstrated that pre-treatment sarcopenia is associated with poorer short- and long-term outcomes in cancer patients. However, most existing reviews focus on specific treatment modalities (e.g., surgery, chemotherapy, or immunotherapy) and lack a systematic integration across cancer types and treatment strategies. Additionally, many reviews have not employed validated tools to assess the methodological quality of the meta-analyses or the certainty of the evidence. Therefore, this umbrella meta-analysis aims to systematically and comprehensively summarize the available meta-analytic evidence from observational studies on sarcopenia and cancer prognosis, and to evaluate the strength and validity of these findings.

## 2 Methods

Our protocol has been registered in PROSPERO (CRD42022383726). The systematic literature search was conducted in accordance with the Preferred Reporting Items for Systematic Reviews and Meta-Analyses (PRISMA) guidelines (Additional file 2) (12).

### 2.1 Search strategy

A systematic search was conducted in PubMed, Web of Science, and Embase from their inception up to October 2025. The search strategy was designed to identify all systematic reviews and meta-analyses investigating the association between sarcopenia and outcomes in cancer patients. A combination of keywords and controlled vocabulary terms (e.g., MeSH in PubMed) was utilized, focusing on the core concepts of “neoplasms,” “sarcopenia,” and “systematic review/meta-analysis.” Detailed search syntax for each database is provided in Supplementary Tables 1, 2. Additionally, the reference lists of all included studies were manually screened to identify additional relevant publications.

### 2.2 Selection of meta-analyses

Two reviewers independently screened the retrieved records by title and abstract, followed by a full-text assessment. Cancers with the incidence or mortality ranking among the major types of cancers were investigated. These cancers included lung cancer, esophageal cancer, gastric cancer, hepatocellular carcinoma, pancreatic carcinoma, hepatic metastasis, colorectal cancer, breast cancer, cervical carcinoma, prostate cancer, renal cell carcinoma and bladder cancer (1). Studies were included if they met the following pre-defined criteria: (a) Published systematic reviews or meta-analyses; (b) Investigated the association between sarcopenia (assessed by any method) and short-term or long-term outcomes in cancer patients; (c) Reported pooled effect estimates with corresponding 95% confidence intervals. Studies that did not perform quantitative synthesis (meta-analysis) and those not published in English were excluded. Any disagreements between the reviewers were resolved through consensus or by consulting a third reviewer.

### 2.3 Data extraction

Data from each eligible meta-analysis were independently extracted by two authors using a standardized form. The extracted information included: first author, publication year, country of origin, number of included studies, total number of patients, specific cancer population, primary anti-cancer treatment modality, health-related outcomes investigated, summary effect measure with 95% confidence interval, I^2^ statistic for heterogeneity, and assessment of publication bias. When a single meta-analysis reported on multiple distinct outcomes, data for each outcome were extracted separately. Furthermore, if multiple meta-analyses investigated the same outcome, the most recently published study with the largest number of included primary studies was prioritized for data extraction to avoid duplication and ensure the synthesis of the most comprehensive evidence.

### 2.4 Quality assessment

The methodological quality of the included meta-analyses was assessed using AMSTAR 2 (A MeaSurement Tool to Assess systematic Reviews 2), while the certainty of evidence for health-related outcomes was evaluated using the GRADE system. AMSTAR 2, a 16-item critical appraisal tool, is an update to the original AMSTAR designed to evaluate systematic reviews of randomized and non-randomized studies (13). Instead of calculating a total score, we categorized the overall confidence in the results into four levels (high, moderate, low, and critically low) based on the presence of critical and non-critical weaknesses in established domains (14–16). Subsequently, the GRADE system was employed to provide a structured assessment of the evidence. Evidence certainty was classified as high, moderate, low, or very low based on five downgrading domains: risk of bias, inconsistency, indirectness, imprecision, and publication bias (17, 18).

## 3 Data analysis

In this umbrella review, we pre-specified stratification by treatment modality (surgical groups and non-surgical groups) to avoid confounding interference. This stratification addresses differences between surgical and systemic cancer treatments that may affect the true prognostic role of sarcopenia.

The data analysis in this study consisted of two components. All analyses were performed using STATA 18.0 software. Given that this umbrella review aimed to integrate studies encompassing diverse cancer types, treatment regimens, and population characteristics, a substantial degree of clinical and methodological heterogeneity was anticipated. Consequently, a random-effects model was uniformly applied to pool the effect sizes (e.g., Hazard Ratio [HR], Odds Ratio [OR]) extracted from these meta-analyses, calculating the summary effect estimates and their 95% confidence intervals (19). This approach was adopted to provide a more conservative and generalizable overall effect estimate. Primary studies were independently searched and supplementary meta-analyses were conducted. For this component, the choice of effect model was determined by the level of statistical heterogeneity: the I^2^ statistic was used to assess between-study heterogeneity, with a random-effects model applied if I^2^ > 50% and a fixed-effects model applied if I^2^ ≤ 50%. All pooled effect estimates are presented with their 95% confidence intervals (20). Publication bias and small study effects were evaluated for each meta-analysis using graphical and statistical methods, specifically the funnel plot and Egger’s test (Egger’s test was performed only when there were at least 10 studies included in the meta-analysis.) (17, 21). The primary studies included in the meta-analyses of this umbrella review, were plotted accordingly. A *P* value of less than 0.10 was considered statistical evidence of the presence of small study effects indicating potential publication bias (21).

## 4 Results

### 4.1 Literature search

**FIGURE 1:**
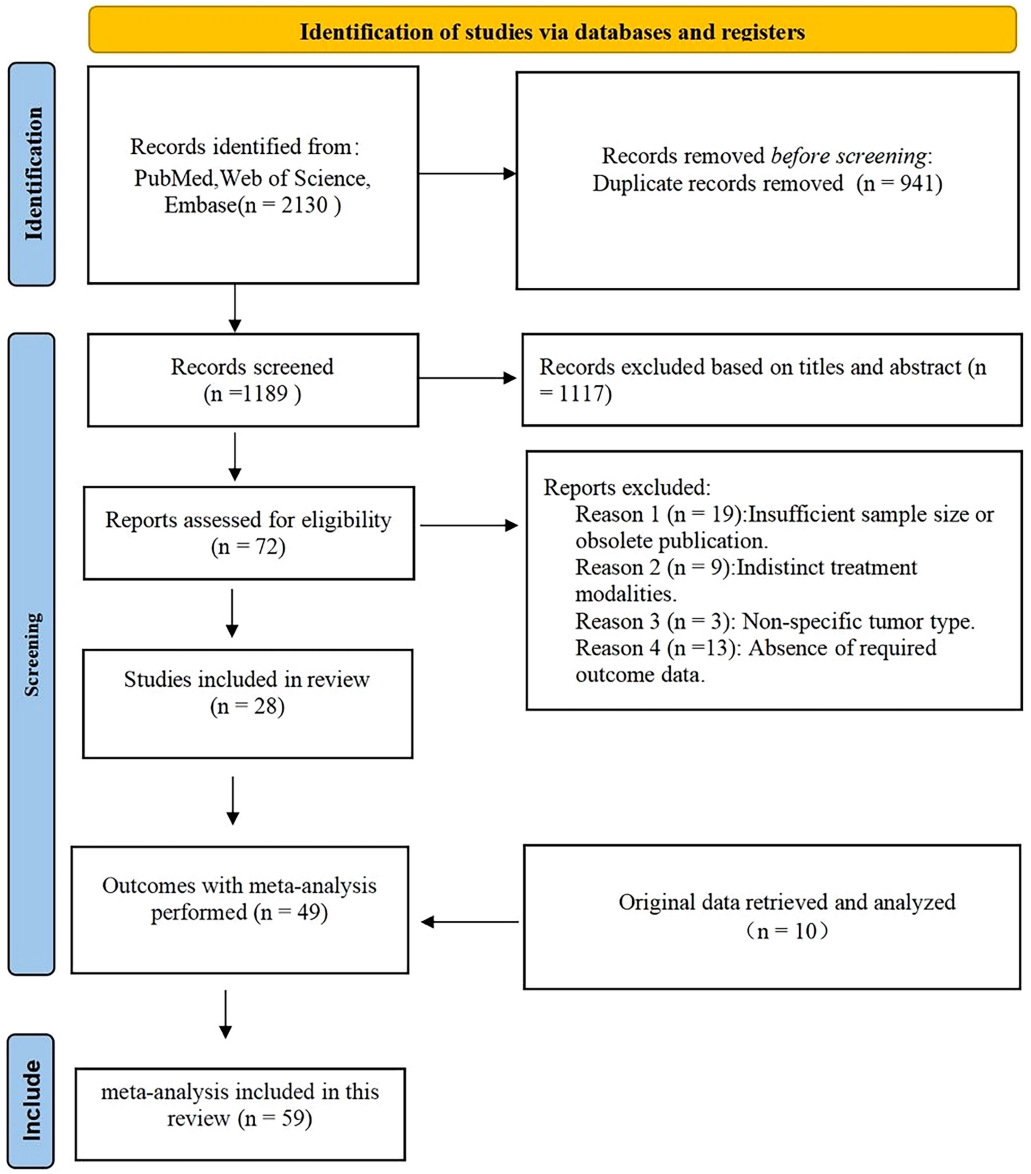
PRISMA flow diagram of the literature review and study selection process.

Of the 2130 records initially identified through database searching (PubMed: 730; Web of Science: 717; Embase: 683), 941 duplicates were removed. Screening of titles and abstracts of the remaining 1,189 records resulted in the exclusion of 1,117 studies. Full-text articles were successfully retrieved for the remaining 72 reports and assessed for eligibility. Following detailed evaluation, 44 reports were excluded with reasons (Supplementary Table 3). Ultimately, 28 studies meeting all inclusion criteria were included in the final review (Figure 1). These comprised: lung cancer (*n* = 3) (22–24), esophageal cancer (*n* = 4) (25–28), gastric cancer (*n* = 2) (10, 29), liver cancer (*n* = 4) (30–33), pancreatic cancer (*n* = 2) (34, 35), liver metastases (*n* = 2) (36, 37), colorectal cancer (*n* = 3) (11, 38, 39), breast cancer (*n* = 1) (40), cervical cancer (*n* = 2) (41, 42), prostate cancer (*n* = 1) (43), renal cell carcinoma (*n* = 1) (44), and bladder cancer (*n* = 3) (45–47), collectively providing data from 49 meta-analyses. Furthermore, we conducted 10 extra meta-analyses to address the gaps in existing literature, these meta-analyses referred to cancers (Supplement Figures 1–10). Finally, a total of 59 meta-analyses were included in pooled assessment.

### 4.2 Characteristics of included studies

The basic characteristics of all studies included in the meta-analysis are summarized in Table 1. These studies encompass diverse cancer populations, including lung cancer, breast cancer, colorectal cancer, among others. The number of original studies included in each meta-analysis ranged from 5 to 45, with a total patient population exceeding 126,575 across all studies. Among them, 18 articles focused on surgical treatment, 9 articles investigated non-surgical first-line treatments, and 1 article addressed both surgical and non-surgical first-line therapies.

**TABLE 1:** Detailed basic information of the literature.

Author, Reference	Country	Included studies	Patients, No.	Cancer population	Anti-cancer treatment	Endpoints of meta-analysis
Liang et al. (22)	China	20	6006	Lung cancer	Surgery	Overall survival, Disease free survival
Kawaguchi et al. (24)	Japan	10	2643	Lung cancer	Surgery	Postoperative complications
Nishimura et al. (23)	USA	9	1010	Lung cancer	Surgery	Postoperative severe complication
Chen et al. (25)	China	26	4515	Esophageal cancer	Surgery	Overall survival, postoperative complications
Jogiat et al. (28)	Canada	21	3966	Esophageal cancer	Surgery	Disease free survival
Wang et al. (27)	China	14	2387	Esophageal cancer	Surgery	Postoperative severe complication
Jogiat et al. (28)	Canada	5	783	Esophageal cancer	First line	Overall survival
Liu et al. (10)	China	42	11,981	Gastric Cancer	Surgery	Overall survival, disease free survival, postoperative complications postoperative severe complication
Meyer et al. (29)	Germany	7	668	Gastric cancer	First line	Overall survival, Progression Free Survival
Kong et al. (30)	China	30	7352	Hepatocellular carcinoma	Surgery	Overall survival, Disease free survival
Zhang et al. (31)	China	12	1774	Hepatocellular carcinoma	Surgery	Postoperative complications
Ji et al. (32)	China	26	4292	Hepatocellular carcinoma	Surgery	Postoperative severe complication
Long et al. (33)	China	12	2559	Hepatocellular carcinoma	First line	Overall survival
Liu et al. (34)	China	23	5888	pancreatic carcinoma	Surgery	Overall survival, Disease free survival, postoperative complications postoperative severe complication
Wang et al. (35)	China	43	6640	pancreatic carcinoma	First line	Overall survival
Waalboer et al. (36)	Netherlands	11	2124	hepatic metastasis	Surgery	Overall survival, Disease free survival
Thormann et al. (37)	Germany	17	3157	hepatic metastasis	Surgery	Postoperative severe complication
Atuiri et al. (11)	USA	17	16,031	Colorectal cancer	Surgery	Overall survival, Disease free survival
van Helsdingen et al. (38)	Netherlands	45	16537	Colorectal cancer	Surgery	Postoperative complications, postoperative severe complication
Meyer et al. (39)	Germany	15	1744	Colorectal cancer	First line	Overall survival, Progression Free Survival
Roberto et al. (40)	Italy	16	6130	Breast cancer	First line	Overall survival, Progression Free Survival
Tian et al. (41)	China	12	1498	Cervical carcinoma	Surgery	Overall survival, Progression Free Survival
Wang et al. (42)	China	14	2020	Cervical carcinoma	First line	Overall survival, Progression Free Survival
de Pablos-Rodríguez et al. (43)	Spain	9	1659	Prostate cancer	First line	Overall survival, Progression Free Survival
Yuxuan et al. (44)	China	18	3591	Renal cell carcinoma	Surgery	Overall survival, Progression Free Survival
Yuxuan et al. (44)	China	18	3591	Renal cell carcinoma	First line	Overall survival
Zeng et al. (45)	China	26	3748	Bladder cancer	Surgery	Overall survival, Disease free survival
Qin and Wu (46)	China	21	4997	Bladder cancer	Surgery	Postoperative complications
Meyer et al. (47)	Germany	11	875	Bladder cancer	First line	Overall survival

**TABLE 2:** Pooled hazard ratios (HRs) with 95% confidence intervals (CIs) and GRADE ratings for primary outcomes.

Author, Reference	Cancer	Included studies (n)	Patients (n)	Metric Effect size HR/*OR* (95% CI)	*p* value	study design	Risk of bias	Inconsistency	Indirectness	Imprecision	Publication bias	Large effect size	Quality
Overall Survival (after surgical treatment)													
Liang et al. (22)	Lung cancer	20	5478	1.67 (1.23–2.26)	< 0.01	non-RCT	Serious	Serious	Not serious	Not serious	Not serious	Not large	⊕○○○ (Very Low)
Chen et al. (25)	Esophageal cancer	16	3025	1.12 (1.04–1.20)	< 0.01	non-RCT	Serious	Not serious	Not serious	Not serious	Not reported	Not large	⊕○○○ (Very Low)
Liu et al. (10)	Gastric cancer	25	7774	1.56 (1.35–1.79)	< 0.001	non-RCT	Serious	Not serious	Not serious	Not serious	Serious	Not large	⊕○○○ (Very Low)
Kong et al. (30)	Hepatocellular carcinoma	28	6916	2.20 (1.88–2.58)	< 0.01	non-RCT	Serious	Not serious	Not serious	Not serious	Not serious	Large	⊕⊕○○ (Low)
Liu et al. (34)	Pancreatic carcinoma	15	4502	1.53 (1.41–1.67)	< 0.00001	non-RCT	Serious	Not serious	Not serious	Not serious	Not serious	Not large	⊕○○○ (Very Low)
Waalboer et al. (36)	Hepatic metastasis	10	1942	1.35 (1.08–1.68)	0.007	non-RCT	Serious	Not serious	Not serious	Not serious	Not serious	Not large	⊕○○○ (Very Low)
Atuiri et al. (11)	Colorectal cancer	16	15640	1.28 (1.04–1.57)	0.02	non-RCT	Serious	Not serious	Not serious	Not serious	Not serious	Not large	⊕○○○ (Very Low)
Tian et al. (41)	Cervical carcinoma	2	439	2.82 (1.36–5.85)	< 0.01	non-RCT	Serious	Not serious	Not serious	Not serious	Not reported	Large	⊕⊕○○ (Low)
Yuxuan et al. (44)	Renal cell carcinoma	6	1917	1.63 (1.19–2.24)	0.2	non-RCT	Serious	Not serious	Not serious	Not serious	Not reported	Not large	⊕○○○ (Very Low)
Zeng et al. (45)	Bladder cancer	18	2519	1.62 (1.43–1.83)	< 0.001	non-RCT	Serious	Not serious	Not serious	Not serious	Not serious	Not large	⊕○○○ (Very Low)
	Breast cancer	3	1000	2.99 (1.60–5.59)	0.001	non-RCT	Serious	Not serious	Not serious	Not serious	undetermined	Large	⊕⊕○○ (Low)
Disease Free Survival (after surgical treatment)													
Liang et al. (22)	Lung cancer	11	3913	1.36 (1.00–1.85)	0.05	non-RCT	Serious	Serious	Not serious	Not serious	Not serious	Not large	⊕○○○ (Very Low)
Jogiat et al. (28)	Esophageal cancer	8	1604	1.73 (1.04–2.87)	0.03	non-RCT	Serious	Not serious	Not serious	Not serious	Not serious	Not large	⊕○○○ (Very Low)
Liu et al. (10)	Gastric cancer	11	4166	1.37 (1.12–1.68)	0.003	non-RCT	Serious	Not serious	Not serious	Not serious	Not reported	Not large	⊕○○○ (Very Low)
Kong et al. (30)	Hepatocellular carcinoma	6	923	1.96 (1.83–2.10)	< 0.01	non-RCT	Serious	Not serious	Not serious	Not serious	Not serious	Not large	⊕○○○ (Very Low)
Liu et al. (34)	Pancreatic carcinoma	6	1185	1.55 (1.31–1.84)	< 0.00001	non-RCT	Serious	Not serious	Not serious	Not serious	Not serious	Not large	⊕○○○ (Very Low)
Waalboer et al. (36)	Hepatic metastasis	7	1505	1.17 (0.94–1.46)	0.15	non-RCT	Serious	Not serious	Not serious	Not serious	Not serious	Not large	⊕○○○ (Very Low)
Atuiri et al. (11)	Colorectal cancer	14	12638	1.23 (1.02–1.49)	0.03	non-RCT	Serious	Not serious	Not serious	Not serious	Not reported	Not large	⊕○○○ (Very Low)
Tian et al. (41)	Cervical carcinoma	2	439	1.96 (1.20–3.19)	0.03	non-RCT	Serious	Not serious	Not serious	Not serious	Not reported	Not large	⊕○○○ (Very Low)
Yuxuan et al. (44)	Renal cell carcinoma	3	1371	1.78 (1.34–2.36)	0.231	non-RCT	Serious	Not serious	Not serious	Not serious	Not reported	Not large	⊕○○○ (Very Low)
Zeng et al. (45)	Bladder cancer	3	428	1.76 (1.21–2.56)	0.003	non-RCT	Serious	Not serious	Not serious	Not serious	Not reported	Not large	⊕○○○ (Very Low)
	Breast cancer	2	423	3.11 (2.29–4.22)	< 0.0001	non-RCT	Serious	Not serious	Not serious	Not serious	undetermined	Large	⊕⊕○○ (Low)
Complications (after surgical treatment)													
Kawaguchi et al. (24)	Lung cancer	7	1538	*1.86 (1.42*–*2.44)*	< 0.00001	non-RCT	Serious	Serious	Not serious	Not serious	Not serious	Not large	⊕○○○ (Very Low)
Chen et al. (25)	Esophageal cancer	20	3765	*1.15 (1.08*–*1.22)*	0.02	non-RCT	Serious	Not serious	Not serious	Not serious	Not serious	Not large	⊕○○○ (Very Low)
Liu et al. (10)	Gastric cancer	21	5907	*2.23 (1.72–2.90)*	< 0.00001	non-RCT	Serious	Not serious	Not serious	Not serious	Not reported	Large	⊕⊕○○ (Low)
Zhang et al. (31)	Hepatocellular carcinoma	12	1774	*1.30 (1.03*–*1.65)*	0.03	non-RCT	Serious	Not serious	Not serious	Not serious	Not serious	Not large	⊕○○○ (Very Low)
Liu et al. (34)	pancreatic carcinoma	6	1863	*1.33 (0.84*–*2.12)*	0.23	non-RCT	Serious	Not serious	Not serious	Not serious	Not reported	Not large	⊕○○○ (Very Low)
van Helsdingen et al. (38)	Colorectal cancer	5	1669	*1.77 (1.15*–*2.72)*	0.009	non-RCT	Serious	Not serious	Not serious	Not serious	Serious	Not large	⊕○○○ (Very Low)
Qin and Wu (46)	Bladder cancer	9	428	*1.77 (1.23*–*2.55)*	NA	non-RCT	Serious	Not serious	Not serious	Not serious	Not serious	Not large	⊕○○○ (Very Low)
	Renal cell carcinoma	2	211	*0.95 (0.45*–*2.01)*	0.904	non-RCT	Serious	Not serious	Not serious	Serious	undetermined	Not large	⊕○○○ (Very Low)
	Breast cancer	2	967	*1.14 (0.80*–*1.63)*	0.554	non-RCT	Serious	Not serious	Not serious	Serious	undetermined	Not large	⊕○○○ (Very Low)
Severe complications (after surgical treatment)													
Nishimura et al. (23)	Lung cancer	1	328	*3.62 (1.01*–*12.95)*	0.05	non-RCT	Serious	Not serious	Not serious	Not serious	Not reported	Large	⊕⊕○○ (Low)
Wang et al. (27)	Esophageal cancer	5	882	*1.30 (0.95*–*1.79)*	0.098	non-RCT	Serious	Not serious	Not serious	Serious	Not serious	Not large	⊕○○○ (Very Low)
Liu et al. (10)	Gastric cancer	19	5569	*1.69 (1.39*–*2.05)*	< 0.00001	non-RCT	Serious	Not serious	Not serious	Not serious	Not reported	Not large	⊕○○○ (Very Low)
Ji et al. (32)	Hepatocellular carcinoma	12	2237	*1.22 (1.02*–*1.47)*	0.033	non-RCT	Serious	Not serious	Not serious	Not serious	Not serious	Not large	⊕○○○ (Very Low)
Liu et al. (34)	Pancreatic carcinoma	18	4877	*1.33 (0.93*–*1.89)*	0.11	non-RCT	Serious	Not serious	Not serious	Not serious	Not serious	Not large	⊕○○○ (Very Low)
Thormann et al. (37)	Hepatic metastasis	6	1188	*1.60 (1.11*–*2.32)*	0.01	non-RCT	Serious	Not serious	Not serious	Not serious	Not serious	Not large	⊕○○○ (Very Low)
van Helsdingen et al. (38)	Colorectal cancer	3	1538	*1.86 (1.37*–*2.53)*	< 0.0001	non-RCT	Serious	Not serious	Not serious	Not serious	Serious	Not large	⊕○○○ (Very Low)
	Renal cell carcinoma	2	243	*2.12 (1.04*–*4.26)*	0.038	non-RCT	Serious	Not serious	Not serious	Not serious	undetermined	Large	⊕⊕○○ (Low)
Overall survival (after first line treatment)													
Jogiat et al. (28)	Esophageal cancer	5	783	1.51 (1.21–1,89)	0.0003	non-RCT	Serious	Not serious	Not serious	Not serious	Not serious	Not large	⊕○○○ (Very Low)
Meyer et al. (29)	Gastric cancer	4	514	1.26 (0.87–1.82)	0.22	non-RCT	Serious	Not serious	Not serious	Serious	Not reported	Not large	⊕○○○ (Very Low)
Long et al. (33)	Hepatocellular carcinoma	9	2127	1.46 (1.30–1.64)	< 0.001	non-RCT	Serious	Not serious	Not serious	Not serious	Not serious	Not large	⊕○○○ (Very Low)
Wang et al. (35)	pancreatic carcinoma	15	2458	1.48 (1.21–1.80)	NA	non-RCT	Serious	Not serious	Not serious	Not serious	Not serious	Not large	⊕○○○ (Very Low)
Meyer et al. (39)	Colorectal cancer	5	577	1.51 (1.20–1.89)	0.0004	non-RCT	Serious	Not serious	Not serious	Not serious	Not reported	Not large	⊕○○○ (Very Low)
Roberto et al. (40)	Breast cancer	9	1791	1.33 (0.97–1.80)	0.07	non-RCT	Serious	Not serious	Not serious	Not serious	Not reported	Not large	⊕○○○ (Very Low)
Wang et al. (42)	Cervical carcinoma	10	1495	1.58 (1.16–2.14)	0.003	non-RCT	Serious	Not serious	Not serious	Not serious	Not serious	Not large	⊕○○○ (Very Low)
de Pablos-Rodríguez et al. (43)	Prostate cancer	7	1318	1.44 (1.23–1.67)	< 0.00001	non-RCT	Serious	Not serious	Not serious	Not serious	Serious	Not large	⊕○○○ (Very Low)
Yuxuan et al. (44)	Renal cell carcinoma	4	342	2.07 (1.07–4.04)	NA	non-RCT	Serious	Not serious	Not serious	Not serious	Not reported	Large	⊕⊕○○ (Low)
Meyer et al. (47)	Bladder cancer	3	205	2.67 (1.80–3.95)	< 0.00001	non-RCT	Serious	Not serious	Not serious	Not serious	Not reported	Large	⊕⊕○○ (Low)
	Lung cancer	9	1331	1.44 (1.10–1.89)	0.007	non-RCT	Serious	Not serious	Not serious	Not serious	Undetermined	Not large	⊕○○○ (Very Low)
Progression free survival (after first line treatment)													
Meyer et al. (29)	Gastric cancer	3	268	1.76 (0.66–4.66)	0.26	non-RCT	Serious	Not serious	Not serious	Serious	Not reported	Not large	⊕○○○ (Very Low)
Meyer et al. (39)	Colorectal cancer	3	440	1.49 (0.94–2.35)	0.09	non-RCT	Serious	Not serious	Not serious	Serious	Not reported	Not large	⊕○○○ (Very Low)
Roberto et al. (40)	Breast cancer	3	357	1.29 (0.79–2.10)	0.32	non-RCT	Serious	Not serious	Not serious	Serious	Not reported	Not large	⊕○○○ (Very Low)
Wang et al. (42)	Cervical carcinoma	5	777	1.63 (1.16–2.29)	0.005	non-RCT	Serious	Not serious	Not serious	Not serious	Not reported	Not large	⊕○○○ (Very Low)
de Pablos-Rodríguez et al. (43)	Prostate cancer	4	825	1.56 (1.29–1.88)	< 0.0001	non-RCT	Serious	Not serious	Not serious	Not serious	Serious	Not large	⊕○○○ (Very Low)
	Esophageal cancer	2	281	1.41 (0.84–2.39)	0.195	non-RCT	Serious	Not serious	Not serious	Serious	Undetermined	Not large	⊕○○○ (Very Low)
	Pancreatic carcinoma	3	393	1.58 (1.05–2.38)	0.027	non-RCT	Serious	Not serious	Not serious	Not serious	Not serious	Not large	⊕○○○ (Very Low)
	Lung cancer	4	787	1.38 (0.79–2.43)	0.262	non-RCT	Serious	Not serious	Not serious	Serious	Undetermined	Not large	⊕○○○ (Very Low)
	Hepatocellular carcinoma	2	138	0.67 (0.42–1.07)	0.097	non-RCT	Serious	Not serious	Not serious	Serious	Undetermined	Not large	⊕○○○ (Very Low)

CI, confidence interval; HR, hazard ratio; non-RCT, non-randomized controlled trial. *This meta-analysis was conducted by the authors specifically for this umbrella review. Italicized values represent odds ratios (ORs), while non-italicized values denote hazard ratios (HRs).

### 4.3 Methodological quality: AMSTAR-2 assessment

Based on AMSTAR-2 criteria, the methodological quality was categorized as follows: 4 were high, 18 were moderate, 4 were low, and 2 were critically low (Supplementary Table 4). The most prevalent methodological deficiency across the assessed domains was the lack of a prior registered protocol, affecting 13 reviews.

### 4.4 Summary of findings and certainty of evidence: GRADE assessment

Key findings from each meta-analysis, including effect estimates, 95% confidence intervals, and *p*-values, were systematically extracted. The quality of evidence for these outcomes was appraised using the GRADE framework. Evidence was subsequently classified as high, moderate, low, or very low. As all evidence originated from observational studies, the initial rating was “low,” and further downgrades were applied due to additional limitations. Consequently, the evidence quality for all 59 outcomes was rated as either low or very low. Detailed results are presented in Table 2.

## 5 Surgical treatment

### 5.1 Association of preoperative sarcopenia with overall survival

**FIGURE 2:**
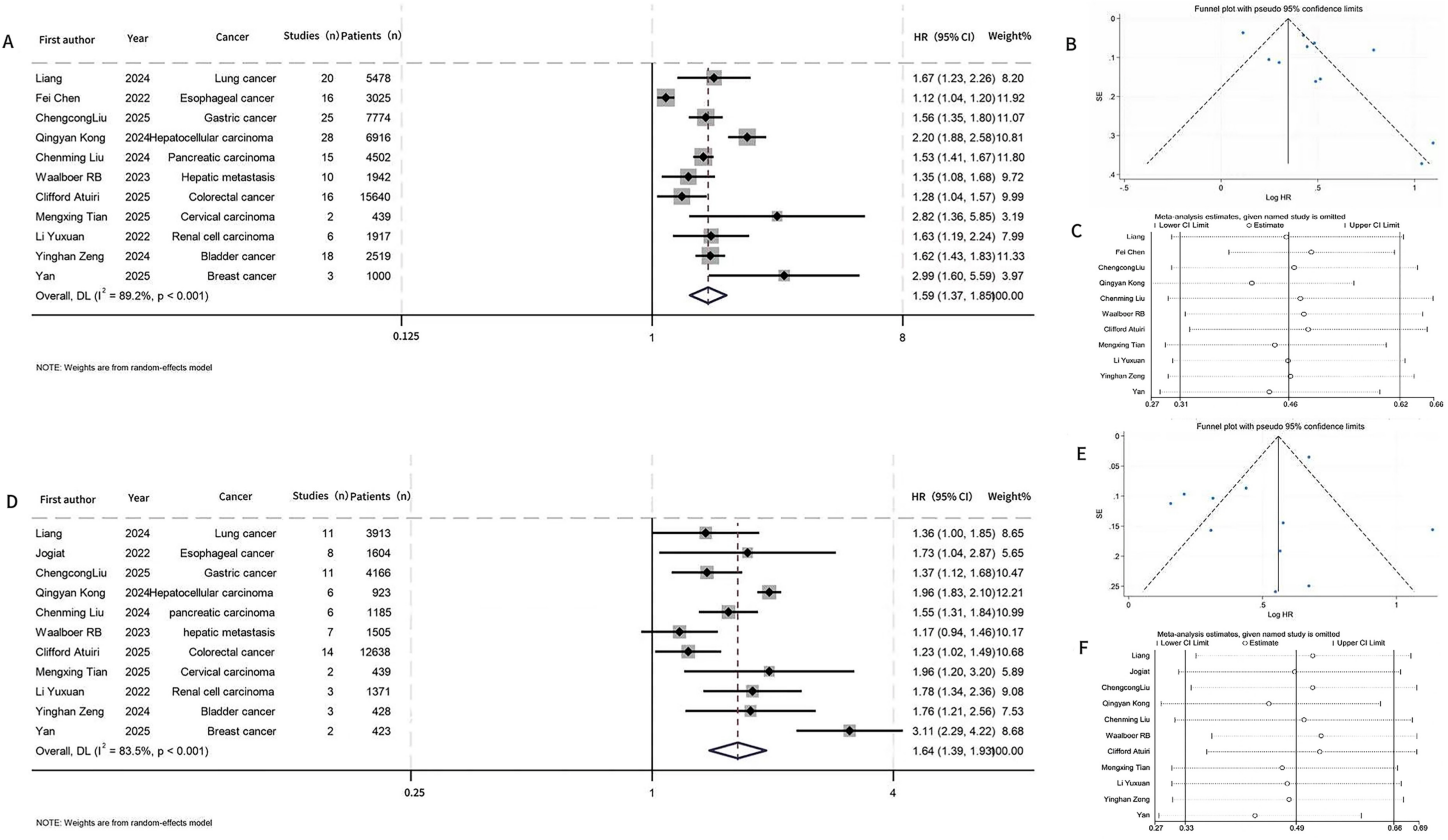
Meta-analysis of survival outcomes after surgery. **(A–C)** Forest plot, funnel plot, and leave-one-out sensitivity analysis for overall survival (OS), respectively. **(D–F)** Forest plot, funnel plot, and leave-one-out sensitivity analysis for disease-free survival (DFS), respectively. The dashed lines are visual dividers only, with no statistical meaning. HR, hazard ratio; CI, confidence interval; non-RCT, non-randomized controlled trial.

While preoperative sarcopenia has been associated with poor OS in individual cancer types, there is a lack of evidence regarding its impact at the pan-cancer level. To address this gap, A meta-analysis of 11 studies (*n* = 50,152) demonstrated that preoperative sarcopenia was significantly associated with reduced overall survival (OS) in cancer patients (pooled HR = 1.59, 95% CI: 1.37–1.85) (Figure 2A). The association was most pronounced in Cervical carcinoma (HR = 2.82) and breast cancer (HR = 2.99). Despite substantial heterogeneity (I^2^ = 89.2%), sensitivity analysis confirmed the robustness of the results, and neither funnel plot inspection nor Egger’s test indicated significant publication bias (*p* = 0.090) (Figures 2B,C).

### 5.2 Association of preoperative sarcopenia with disease-free survival

While the impact of preoperative sarcopenia on disease-free survival (DFS) has been explored in individual malignancies, its collective significance at a pan-cancer level has not been systematically established. A meta-analysis of 11 additional studies (*n* = 28,595) specifically evaluated the impact of preoperative sarcopenia on disease-free survival (DFS). Sarcopenia was significantly associated with reduced DFS (pooled HR = 1.64, 95% CI: 1.39–1.93) (Figure 2D). This association remained consistent despite substantial heterogeneity among studies (I^2^ = 83.5%). Furthermore, Egger’s test indicated no significant publication bias (*p* = 0.305) (Figure 2E), and sensitivity analyses confirmed the robustness of the pooled results (Figure 2F).

### 5.3 Association of preoperative sarcopenia with overall complications

**FIGURE 3:**
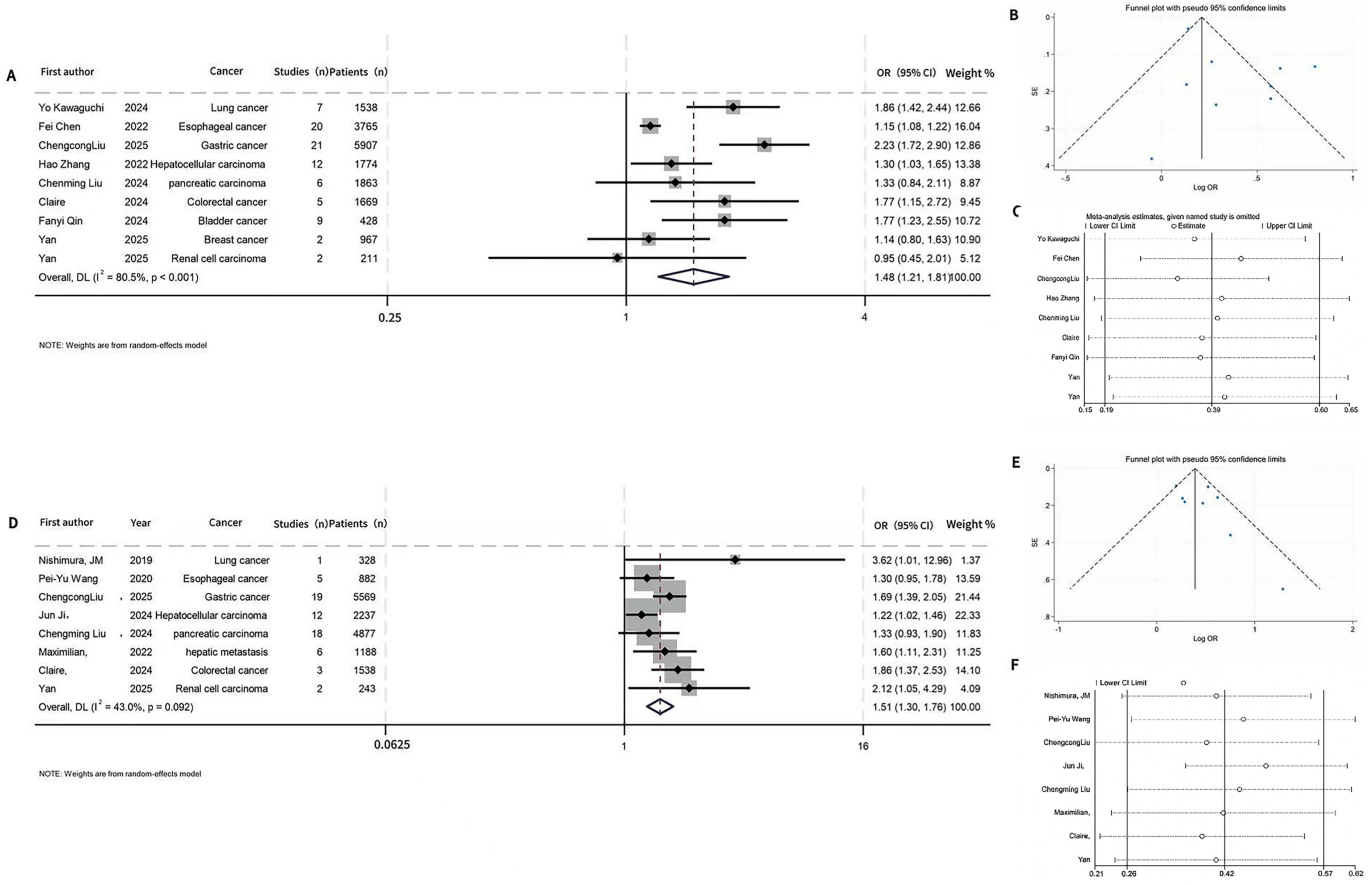
Meta-analysis of postoperative complications. **(A–C)** Forest plot, funnel plot, and leave-one-out sensitivity analysis for overall postoperative complications, respectively. **(D–F)** Forest plot, funnel plot, and leave-one-out sensitivity analysis for major postoperative complications, respectively. The dashed lines are visual dividers only, with no statistical meaning. HR, hazard ratio; CI, confidence interval; non-RCT, non-randomized controlled trial.

Although preoperative sarcopenia is a recognized risk factor for postoperative complications in specific surgical cohorts, a comprehensive evaluation of this association across diverse cancer types remains lacking. A meta-analysis of 9 studies (*n* = 18,122) on short-term postoperative outcomes revealed that preoperative sarcopenia significantly increased the risk of overall complications (pooled OR = 1.48, 95% CI: 1.21–1.81) (Figure 3A). The risk was most prominent in gastric cancer (OR = 2.23) and lung cancer (OR = 1.86). Despite high heterogeneity (I^2^ = 80.5%), no significant publication bias was detected (Egger’s test *p* = 0.077) (Figure 3B), and sensitivity analysis confirmed the robustness of the pooled estimate (Figure 3C).

### 5.4 Association of preoperative sarcopenia with major complications

While many studies have reported a correlation between sarcopenia and major postoperative complications in single-organ cancers, evidence synthesizing this risk from a pan-cancer perspective is currently limited. Analysis of major complications (8 studies, *n* = 16,882) established preoperative sarcopenia as a significant risk factor (pooled OR = 1.51, 95% CI: 1.30–1.76) (Figure 3D). Despite moderate heterogeneity (I^2^ = 43.0%), no significant publication bias was detected (Egger’s test *p* = 0.202) (Figure 3E), and sensitivity analysis confirmed the robustness of this finding (Figure 3F).

## 6 First-line treatment

### 6.1 Association between pretreatment sarcopenia and overall survival

**FIGURE 4:**
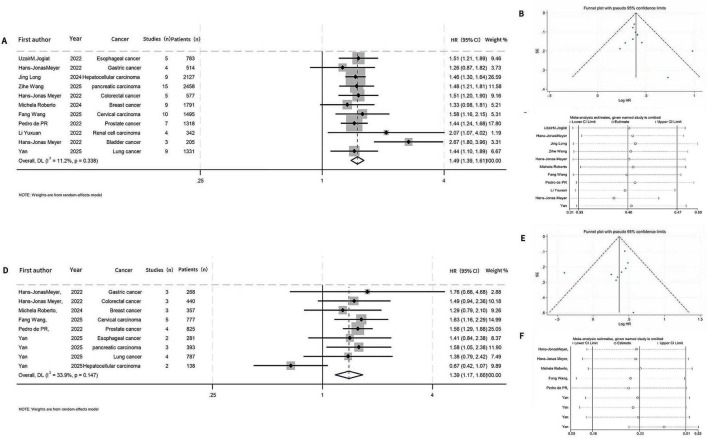
Meta-analysis of survival outcomes after first-line therapy. **(A–C)** Forest plot, funnel plot, and leave-one-out sensitivity analysis for overall survival (OS), respectively. **(D–F)** Forest plot, funnel plot, and leave-one-out sensitivity analysis for progression-free survival (PFS), respectively. The dashed lines are visual dividers only, with no statistical meaning. HR, hazard ratio; CI, confidence interval; non-RCT, non-randomized controlled trial.

Pretreatment sarcopenia has been linked to poor overall survival (OS) in patients receiving first-line therapy for certain cancers; however, its prognostic value has yet to be validated through a large-scale pan-cancer synthesis. A meta-analysis of 11 studies (*n* = 12,945) on patients receiving first-line therapy demonstrated that pretreatment sarcopenia is a significant independent risk factor for poorer overall survival (pooled HR = 1.49, 95% CI: 1.39–1.61) (Figure 4A). The association was most pronounced in bladder cancer (HR = 2.67) and renal cell carcinoma (HR = 2.07). The analysis showed low heterogeneity (I^2^ = 11.2%) and no significant publication bias (Egger’s test *p* = 0.204) (Figure 4B). Sensitivity analysis confirmed the robustness of the pooled result (Figure 4C).

### 6.2 Association of pretreatment sarcopenia with progression-free survival

Despite evidence suggesting that sarcopenia affects progression-free survival (PFS) in individual cancer populations undergoing first-line treatment, there is a clear need for a systematic pan-cancer assessment to confirm this trend. A meta-analysis of 9 studies (*n* = 4,266) demonstrated that pretreatment sarcopenia is a significant independent risk factor for reduced progression-free survival in patients receiving first-line therapy (pooled HR = 1.39, 95% CI: 1.17–1.66) (Figure 4D). The analysis revealed low heterogeneity (I^2^ = 33.9%) and no significant publication bias (Egger’s test *p* = 0.409) (Figure 4E). Sensitivity analysis confirmed the robustness of this finding (Figure 4F).

## 7 Immunotherapy

In the context of immunotherapy, sarcopenia has emerged as a significant predictor of adverse outcomes, based on findings from the limited number of eligible meta-analyses included in this umbrella review. This is particularly evident in lung cancer, where sarcopenia is strongly associated with inferior survival (1- and 2-year OS OR = 2.44/1.60), reduced progression-free survival (1- and 2-year PFS OR = 3.43/2.06), and poorer treatment response, as reflected by lower objective response rates (ORR OR = 2.22) and disease control rates (DCR OR = 3.15) (48). Importantly, this negative prognostic impact extends beyond lung cancer, with sarcopenia also predicting significantly shorter OS and PFS in patients with hepatocellular carcinoma and gastric cancer (49). Collectively, the evidence establishes sarcopenia as a robust, independent prognostic factor for diminished survival and therapeutic efficacy in patients undergoing immune checkpoint inhibitor therapy.

## 8 Discussion

This umbrella review systematically synthesized the evidence regarding the impact of sarcopenia on therapeutic outcomes across the most prevalent cancer types by global incidence, with 59 meta-analyses included. Sarcopenia was demonstrated to adversely influence outcomes of treatment modalities and should be highlighted in management of cancer patients.

Multiple meta-analyses have established sarcopenia as a predictor of adverse outcomes in cancer patients (25, 34, 42, 47). We investigated common malignancies with high incidence or mortality and prespecified a stratified analysis (surgical groups and non-surgical groups). The results consistently demonstrated that sarcopenia is independently associated with poor prognosis, significantly reducing overall survival and markedly increasing the incidence of postoperative complications among surgical patients. Notably, sarcopenia further elevates the risk of both overall and major postoperative complications across multiple cancer subtypes. In addition, consistent prognostic impacts of sarcopenia are also observed in patients with lung cancer, hepatocellular carcinoma and gastric cancer receiving immunotherapy, manifesting as inferior survival outcomes and poorer treatment response. Furthermore, for cancer types with relatively lower incidence not covered in this paper, numerous studies have also indicated that sarcopenia leads to adverse outcomes. For instance, a large meta-analysis published in 2025 showed that sarcopenia is associated with poor prognosis in endometrial cancer (50). In hematological malignancies, sarcopenia serves as an independent predictor of overall survival in patients with diffuse large B-cell lymphoma treated with chemotherapy (51). The clinical significance of sarcopenia is not limited to oncology. In cardiovascular and cerebrovascular diseases, such as heart failure, sarcopenia is associated with higher all-cause mortality (52). In patients with liver cirrhosis, the presence of sarcopenia is associated with poorer prognosis and a higher risk of complications (53). Collectively, this evidence suggests that sarcopenia may act as a common cross-disease risk factor, playing a critical role in the development and outcomes of various chronic conditions. Therefore, it warrants greater attention in clinical assessment and intervention.

Our conclusions reinforce those of previous meta-analyses. Sensitivity analysis verified the statistical robustness of our pooled prognostic estimates, confirming that the overall findings were not markedly influenced by any single included meta-analysis. Despite the observational nature of the evidence, the consistency and magnitude of this association across diverse cancers and treatment modalities underscore its profound clinical importance. The mechanisms by which pretreatment muscle status influences treatment outcomes remain unclear. Skeletal muscle serves not only as a vital endocrine and immunomodulatory organ, whose secreted myokines (e.g., IL-6, IL-7, IL-15) regulate immune function, with muscle loss leading to immune dysfunction, potentially explaining the compounded risk observed when sarcopenia coexists with impaired immune-nutritional status (54–56), but also as a crucial peripheral reservoir of protein and energy (57, 58). Under metabolic stress conditions such as major surgery, severe infection, or critical burns, skeletal muscle is rapidly catabolized to supply amino acids, particularly glutamine, to the liver, intestines, and immune system (57, 59). Even with adequate exogenous amino acid provision, the perioperative period is often accompanied by skeletal muscle catabolism, especially within the pathophysiological contexts of immune response, inflammatory reactions, and tissue repair (59, 60). Furthermore, a synergistic effect between sarcopenia and systemic inflammation—termed the “sarcopenia-inflammation axis”—has been proposed as a common pathway accelerating disease progression across multiple cancers (61).

Sarcopenia is a prevalent pathological condition with adverse effects, and its association with poor clinical outcomes has achieved broad consensus in the academic community. Current clinical practice increasingly recognizes that improving muscle status before treatment is crucial for enhancing prognosis, increasing treatment tolerance, and reducing treatment-related adverse events. Therefore, systematically screening for sarcopenia before treatment and establishing standardized assessment guidelines are of great importance. Future evaluations should adopt a multidimensional approach, considering not only muscle quantity but also factors such as muscle quality (62). Particularly for non-emergency patients, dynamically monitoring muscle status before, during, and after treatment can provide critical guidance for clinical decision-making. For patients already experiencing muscle loss, proactively implementing comprehensive measures before treatment—including nutritional support, exercise training, and pharmacological interventions when necessary—may be key for improving overall survival and quality of life in this vulnerable population (63). Interventions and management of sarcopenia may represent potential strategies for improving treatment outcomes of multiple treatments and clinical prognosis, which warrants further validation in clinical trials.

## 9 Limitations

Several limitations of the present study should also be acknowledged. First, the methods and diagnostic thresholds for defining sarcopenia varied considerably across the included primary studies, potentially affecting the comparability and consistency of the findings (62, 64–66). Second, the predominantly retrospective design of the evidence presents challenges in controlling for potential confounders and increases the risk of bias (67, 68). Third, the existing evidence primarily focuses on establishing the association between sarcopenia and poor prognosis, while high-quality research investigating whether interventions to improve sarcopenia can directly translate into survival benefits or improvements in other clinical outcomes remains limited. These limitations highlight the necessity for future prospective studies utilizing standardized sarcopenia assessments to validate our findings and clarify whether sarcopenia serves as a causal risk factor or merely a marker of advanced disease.

## 10 Conclusion

This umbrella meta-analysis confirms that sarcopenia significantly impacts both short-and long-term prognosis in cancer patients across various treatment modalities, including surgery, first-line therapy, and immunotherapy. Its adverse effects are reflected in an increased risk of complications and reduced overall survival. These findings emphasize the importance of sarcopenia assessment and its integration into clinical practice prior to cancer treatment. Future research should focus on validating whether targeted nutritional and physical interventions can reverse sarcopenia and succinctly improve cancer survival rate.

## Data Availability

The original contributions presented in this study are included in this article/Supplementary material, further inquiries can be directed to the corresponding authors.
