# Supplementary material for: Highlighting sarcopenia management in cancer treatments: evidence from umbrella meta-analysis
Source: Front Nutr. 2026 Jul 16;13:1831634. doi: 10.3389/fnut.2026.1831634 (PMC13421404; doi:10.3389/fnut.2026.1831634)
Supplement: Supplementary file 1 [file Table_1.docx]

***Supplementary Information***

**Table S1:** PubMed search strategy (with limit for systematic reviews and meta-analysis)

**Table S2:** PubMed Search strategy (without limit for systematic reviews and meta-analysis)

**Table S3:** Included and excluded systematic reviews and meta-analyses, with reasons

**Table S4:** AMSTAR 2 quality assessment of meta-analyses included

**Figure S1:** Overall Survival After First-Line Therapy in Lung Cancer.

**Figure S2:** Progression Free Survival After First-Line Therapy in Lung Cancer.

**Figure S3:** Progression Free Survival After First-Line Therapy in Esophageal Cancer.

**Figure S4:** Progression Free Survival After First-Line Therapy in Hepatocellular Carcinoma.

**Figure S5:** Progression Free Survival After First-Line Therapy in Pancreatic Carcinoma.

**Figure S6:** Overall Survival After Surgery in Breast Cancer.

**Figure S7:** Disease Free Survival After Surgery in Breast Cancer.

**Figure S8:** Postoperative Complications in Breast Cancer.

**Figure S9:** Postoperative Complications in Renal Cell Carcinoma.

**Figure S10:** Postoperative Major Complications in Renal Cell Carcinoma.

**Supplementary Table S1**: **PubMed search strategy (with limit for systematic reviews and meta-analysis)**

| Set | Search Strategy |
| --- | --- |
| Limit: Language | (English[lang]) |
| Limit: Exclude animal only | NOT ("Animals"[Mesh] NOT ("Animals"[Mesh] AND "Humans"[Mesh])) |
| Limit: Exclude child only | NOT (("infant"[Mesh] OR "child"[mesh] OR "adolescent"[mh]) NOT (("infant"[Mesh] OR "child"[mesh] OR "adolescent"[mh]) AND "adult"[Mesh])) |
| Limit: Publication Type Include Systematic Reviews/Meta-Analyses | AND (systematic[sb] OR meta-analysis[pt] OR “systematic review”[tiab] OR “systematic literature review”[tiab] OR metaanalysis[tiab] OR "meta analysis"[tiab] OR metanalyses[tiab] OR "meta analyses"[tiab] OR "pooled analysis"[tiab] OR “pooled analyses”[tiab] OR "pooled data"[tiab]) |
| Limit: Publication Type Exclude Systematic Reviews/Meta-Analyses | NOT (“comment”[Publication Type] OR “editorial”[Publication Type]) |
| Sarcopenic | AND(“sarcopenic”[tiab] OR “muscle”[tiab] OR “skeletal muscle loss”[tiab] OR “muscle wasting”[tiab] OR “muscle atrophy”[tiab] OR “muscle function impairment”[tiab]) |
| Cancer | AND ("Lung Cancer"[mh] OR "Esophageal Cancer"[mh] OR "OR "Gastric Cancer"[mh] OR "Hepatocellular carcinoma"[mh] OR "pancreatic carcinoma"[mh] OR "hepatic metastasir"[mh]OR "Colorectal cancer"[mh] OR "Breast cancer"[mh] OR "Cervical carcinoma"[mh] OR "Prostate cancer"[mh] OR "Renal cell carcinoma"[mh] OR "Bladder cancer"[mh]) |

| Set | Search Strategy |
| --- | --- |
| Limit: Language | (English[lang]) |
| Limit: Exclude animal only | NOT ("Animals"[Mesh] NOT ("Animals"[Mesh] AND "Humans"[Mesh])) |
| Limit: Exclude child only | NOT (("infant"[Mesh] OR "child"[mesh] OR "adolescent"[mh]) NOT (("infant"[Mesh] OR "child"[mesh] OR "adolescent"[mh]) AND "adult"[Mesh])) |
| Sarcopenic | AND(“sarcopenic”[tiab] OR “muscle”[tiab] OR “skeletal muscle loss”[tiab] OR “muscle wasting”[tiab] OR “muscle atrophy”[tiab] OR “muscle function impairment”[tiab]) |
| Cancer | AND ("Lung Cancer"[mh] OR "Esophageal Cancer"[mh] OR "OR "Gastric Cancer"[mh] OR "Hepatocellular carcinoma"[mh] OR "pancreatic carcinoma"[mh] OR "hepatic metastasir"[mh]OR "Colorectal cancer"[mh] OR "Breast cancer"[mh] OR "Cervical carcinoma"[mh] OR "Prostate cancer"[mh] OR "Renal cell carcinoma"[mh] OR "Bladder cancer"[mh]) |

**Supplementary Table S2**: **PubMed Search strategy (without limit for systematic reviews and meta-analysis)**

**Supplementary Table S3: Included and excluded systematic reviews and meta-analyses, with reasons**

| Author | Year | Title | include/exclude | Excluded Cause |
| --- | --- | --- | --- | --- |
| Judith Buentzel | 2019 | Sarcopenia as Prognostic Factor in Lung Cancer Patients: A Systematic Review and Meta-analysis | exclude | Indistinct treatment modalities. |
| Sandra Jensen | 2023 | Sarcopenia and loss of muscle mass in patients with lung cancer undergoing chemotherapy treatment: a systematic review and meta-analysis | exclude | Absence of required outcome data |
| Liang | 2024 | Both thoracic and abdominal muscles can be used as effective indicators for sarcopenia in patients with lung cancer undergoing surgery: A systematic review and meta-analysis. | include | NA |
| Nishimura | 2019 | Computed Tomography-Assessed Skeletal Muscle Mass as a Predictor of Outcomes in Lung Cancer Surgery | include | NA |
| Ming Yang | 2019 | Prognostic Value of Sarcopenia in Lung Cancer: A Systematic Review and Meta-analysis | exclude | Indistinct treatment modalities. |
| Han-Yu Deng | 2019 | Sarcopenia is an independent unfavorable prognostic factor of non-small cell lung cancer after surgical resection: A comprehensive systematic review and meta-analysis. | exclude | Insufficient sample size or obsolete publication. |
| Judith Buentzel | 2019 | Sarcopenia as Prognostic Factor in Lung Cancer Patients: A Systematic Review and Meta-analysis | exclude | Absence of required outcome data |
| Chattarin Pumtako | 2025 | Prevalence and Prognostic Value of Global Leadership Initiative on Malnutrition (GLIM) Phenotypic Cachexia Criteria in Cancer Patients: A Systematic Review and Meta-Analysis | exclude | Non-specific tumor type |
| M J J Voorn | 2022 | Associations between pretreatment nutritional assessments and treatment complications in patients with stage I-III non-small cell lung cancer: A systematic review | exclude | Absence of required outcome data |
| Kawaguchi | 2021 | Does sarcopenia affect postoperative short- and long-term outcomes in patients with lung cancer?-a systematic review and meta-analysis. | include | NA |
| Pei-Yu Wang | 2020 | Sarcopenia and Short-Term Outcomes After Esophagectomy: A Meta-analysis | include | NA |
| Pei-Yu Wang | 2022 | Skeletal muscle wasting during neoadjuvant therapy as a prognosticator in patients with esophageal and esophagogastric junction cancer: A systematic review and meta-analysis. | exclude | Absence of required outcome data |
| Amanda Park | 2023 | The impact of sarcopenia on esophagectomy for cancer: a systematic review and meta-analysis | exclude | Absence of required outcome data |
| Sheng-Bo Jin | 2021 | The Impact of Preoperative Sarcopenia on Survival Prognosis in Patients Receiving Neoadjuvant Therapy for Esophageal Cancer: A Systematic Review and Meta-Analysis | exclude | Indistinct treatment modalities. |
| Dimitrios | 2020 | The impact of preoperative sarcopenia on postoperative complications following esophagectomy for esophageal neoplasia: a systematic review and meta-analysis | exclude | Insufficient sample size or obsolete publication. |
| Fei Chen | 2022 | Impact of preoperative sarcopenia on postoperative complications and survival outcomes of patients with esophageal cancer: a meta-analysis of cohort studies | include | NA |
| Han-Yu Deng | 2019 | Preoperative sarcopenia is a predictor of poor prognosis of esophageal cancer after esophagectomy: a comprehensive systematic review and meta-analysis | exclude | Insufficient sample size or obsolete publication. |
| P R Boshier | 2018 | Assessment of body composition and sarcopenia in patients with esophageal cancer: a systematic review and meta-analysis | exclude | Insufficient sample size or obsolete publication. |
| Uzair M Jogiat | 2022 | Sarcopenia Determined by Skeletal Muscle Index Predicts Overall Survival, Disease-free Survival, and Postoperative Complications in Resectable Esophageal Cancer: A Systematic Review and Meta-analysis. | include | NA |
| Li Yao | 2022 | Prognostic Value of Pretreatment Skeletal Muscle Mass Index in Esophageal Cancer Patients: A Meta-Analysis | exclude | Insufficient sample size or obsolete publication. |
| Uzair M Jogiat | 2022 | Sarcopenia reduces overall survival in unresectable oesophageal cancer: a systematic review and meta-analysis | include | NA |
| Sivesh | 2019 | Body composition assessment and sarcopenia in patients with gastric cancer: a systematic review and meta-analysis | exclude | Insufficient sample size or obsolete publication. |
| Yanjiao Shen | 2017 | The impact of frailty and sarcopenia on postoperative outcomes in older patients undergoing gastrectomy surgery: a systematic review and meta-analysis | exclude | Insufficient sample size or obsolete publication. |
| Alicia S Borggreve | 2020 | The Predictive Value of Low Muscle Mass as Measured on CT Scans for Postoperative Complications and Mortality in Gastric Cancer Patients: A Systematic Review and Meta-Analysis | exclude | Insufficient sample size or obsolete publication. |
| Masayoshi Terayama | 2023 | Long-term impact of sarcopenia in older patients undergoing gastrectomy for gastric cancer: a systematic review and meta-analysis | exclude | Insufficient sample size or obsolete publication. |
| Chenyang Zhan | 2025 | Postoperative skeletal muscle loss as a prognostic indicator of clinical outcomes in patients with gastric cancer: a systematic review and meta-analysis | exclude | Insufficient sample size or obsolete publication. |
| Chengcong Liu | 2025 | The impact of preoperative skeletal muscle mass index-defined sarcopenia on postoperative complications and survival in gastric cancer: An updated meta-analysis | include | NA |
| Marieh | 2023 | The association between skeletal muscle mass index (SMI) and survival after gastrectomy: A systematic review and meta-analysis of cohort studies | exclude | Absence of required outcome data |
| Fei Chen | 2022 | Impact of preoperative sarcopenia on postoperative complications and prognosis of gastric cancer resection: A meta-analysis of cohort studies | exclude | Insufficient sample size or obsolete publication. |
| Zhengdao Yang | 2018 | Predictive Value of Preoperative Sarcopenia in Patients with Gastric Cancer: a Meta-analysis and Systematic Review | exclude | Insufficient sample size or obsolete publication. |
| Rinninella | 2019 | Musclemass,assessed at diagnosis by L3-CT scan as a prognostic marker of clinical outcomes in patients with gastric cancer: A systematic review and meta-analysis | exclude | Indistinct treatment modalities. |
| Hans-Jonas Meyer | 2022 | Sarcopenia as a Prognostic Marker for Survival in Gastric Cancer Patients Undergoing Palliative Chemotherapy. A Systematic Review and Meta Analysis | include | NA |
| Alexandros | 2024 | The Effects of Sarcopenia on Overall Survival and Postoperative Complications of Patients Undergoing Hepatic Resection for Primary or Metastatic Liver Cancer: A Systematic Review and Meta-Analysis | exclude | Absence of required outcome data |
| Chuan Jiang | 2022 | Association between sarcopenia and prognosis of hepatocellular carcinoma: A systematic review and meta-analysis | exclude | Indistinct treatment modalities. |
| Rongqiang Liu | 2023 | High intramuscular adipose tissue content associated with prognosis and postoperative complications of cancers | exclude | Absence of required outcome data |
| Jing Long | 2024 | The predictive value of sarcopenia and myosteatosis in trans-arterial (chemo)-embolization treated HCC patients | include | NA |
| Anrong Wang | 2024 | Impact of body composition on the prognosis of hepatocellular carcinoma patients treated with transarterial chemoembolization: A systematic review and meta-analysis | exclude | Insufficient sample size or obsolete publication. |
| Qingyan Kong | 2024 | The Impact of Imaging-Diagnosed Sarcopenia on Long-term Prognosis After Curative Resection for Hepatocellular Carcinoma: A Systematic Review and Meta-analysis | include | NA |
| Hao Zhang | 2023 | Sarcopenia Predicts Prognosis of Patients Undergoing Liver Resection for Hepatocellular Carcinoma: A Systematic Review and Meta-Analysis | include | NA |
| Jun Ji | 2024 | Impact of imaging-diagnosed sarcopenia on outcomes in patients with biliary tract cancer after surgical resection: a systematic review and meta-analysis | include | NA |
| Ruben B Waalboer | 2022 | Sarcopenia and long-term survival outcomes after local therapy for colorectal liver metastasis: a meta-analysis | include | NA |
| D Wagner | 2023 | Value of sarcopenia in the resection of colorectal liver metastases-a systematic review and meta-analysis | exclude | Absence of required outcome data |
| Maximilian | 2022 | Low skeletal muscle mass and post-operative complications after surgery for liver malignancies: a meta-analysis | include | NA |
| Chenming Liu | 2024 | Association between preoperative sarcopenia and prognosis of pancreatic cancer after curative-intent surgery: a updated systematic review and meta-analysis | include | NA |
| Elisa | 2021 | The Prognostic Value of Low Muscle Mass in Pancreatic Cancer Patients: A Systematic Review and Meta-Analysis | exclude | Insufficient sample size or obsolete publication. |
| Khalil | 2024 | Impact of Cachexia on Chemotherapy Efficacy and Survival in Pancreatic Cancer: A Systematic Review and Meta-Analysis | exclude | Absence of required outcome data |
| Zihe Wang | 2025 | Looking at or beyond the tumor - a systematic review and meta-analysis of quantitative imaging biomarkers predicting pancreatic cancer prognosis | include | NA |
| Li Yang | 2023 | Prognostic value of pretreatment skeletal muscle index in pancreatic carcinoma patients: A meta-analysis | exclude | Insufficient sample size or obsolete publication. |
| Guangwei Sun | 2018 | Can sarcopenia be a predictor of prognosis for patients with non-metastatic colorectal cancer? A systematic review and meta-analysis | exclude | Insufficient sample size or obsolete publication. |
| Qiutong Su | 2024 | Predictive role of preoperative sarcopenia for long-term survival in rectal cancer patients: A meta-analysis | exclude | Insufficient sample size or obsolete publication. |
| Jie He | 2023 | Sarcopenia as a prognostic indicator in colorectal cancer: an updated meta-analysis | exclude | Insufficient sample size or obsolete publication. |
| I Drami | 2021 | Body Composition and Dose-limiting Toxicity in Colorectal Cancer Chemotherapy Treatment; a Systematic Review of the Literature. Could Muscle Mass be the New Body Surface Area in Chemotherapy Dosing? | exclude | Absence of required outcome data |
| Clifford Atuiri | 2025 | Pretreatment Skeletal Muscle Index and Survival Outcomes in Non-Metastatic Colorectal Cancer: A Systematic Review and Meta-Analysis | include | NA |
| Claire | 2024 | Association of computed tomography-derived body composition and complications after colorectal cancer surgery: A systematic review and meta-analysis | include | NA |
| Hans-Jonas Meyer | 2022 | Prognostic Role of Low-Skeletal Muscle Mass on Staging Computed Tomography in Metastasized Colorectal Cancer: A Systematic Review and Meta-Analysis | include | NA |
| G F P Aleixo | 2019 | Muscle composition and outcomes in patients with breast cancer: meta-analysis and systematic review | exclude | Indistinct treatment modalities. |
| Xiao-Ming Zhang | 2020 | Sarcopenia as a predictor of mortality in women with breast cancer: a meta-analysis and systematic review | exclude | Indistinct treatment modalities. |
| Michela Roberto | 2024 | Sarcopenia in Breast Cancer Patients: A Systematic Review and Meta-Analysis | include | NA |
| Fang Wang | 2025 | The Prognostic Value of Sarcopenia in Clinical Outcomes in Cervical Cancer: A Systematic Review and Meta-Analysis | include | NA |
| Allanson | 2020 | A systematic review and meta-analysis of sarcopenia as a prognostic factor in gynecological malignancy | exclude | Non-specific tumor type |
| Mengxing Tian | 2025 | Pretreatment Computed Tomography-Defined Sarcopenia, Treatment-Associated Muscle Loss, and Survival in Patients With Cervical Cancer: A Systematic Review and Meta-Analysis | include | NA |
| Pedro de | 2022 | Prognostic Impact of Sarcopenia in Patients with Advanced Prostate Carcinoma: A Systematic Review | include | NA |
| Hans-Jonas Meyer | 2022 | CT-defined low-skeletal muscle mass as a prognostic marker for survival in prostate cancer: A systematic review and meta-analysis | exclude | Insufficient sample size or obsolete publication. |
| Milena | 2023 | The trajectory of sarcopenia following diagnosis of prostate cancer: A systematic review and meta-analysis | exclude | Indistinct treatment modalities. |
| Hans-Jonas Meyer | 2022 | Low Skeletal Muscle Mass Predicts Relevant Outcomes in Palliative Urological Oncology: A Systematic Review and Meta-Analysis | exclude | Absence of required outcome data |
| Shuluan Li | 2021 | Prognostic Impact of Sarcopenia on Clinical Outcomes in Malignancies Treated With Immune Checkpoint Inhibitors: A Systematic Review and Meta-Analysis | exclude | Non-specific tumor type |
| Li Yuxuan | 2022 | The role of sarcopenia in treatment-related outcomes in patients with renal cell carcinoma: A systematic review and meta-analysis | include | NA |
| Xu Hu | 2020 | Sarcopenia predicts prognosis of patients with renal cell carcinoma: A systematic review and meta-analysis | exclude | Indistinct treatment modalities. |
| Ornaghi | 2021 | The impact of preoperative nutritional status on post-surgical complication and mortality rates in patients undergoing radical cystectomy for bladder cancer: a systematic review of the literature | exclude | Absence of required outcome data |
| Fanyi Qin | 2024 | Impact of sarcopenia on outcomes of bladder cancer undergoing radical cystectomy: A systematic review and meta-analysis | include | NA |
| Yinghan Zeng | 2024 | Prognostic Effects of Sarcopenia on Patients with Bladder Cancer: A Systematic Review and Meta-Analysis | include | NA |
| Hans-Jonas Meyer | 2022 | Low Skeletal Muscle Mass Predicts Relevant Outcomes in Palliative Urological Oncology: A Systematic Review and Meta-Analysis | include | NA |

**Supplementary Table S4. AMSTAR 2 quality assessment of meta-analyses included**

| **Author, Year** | **AMSTAR 2 items ^a, c^** | | | | | | | | | | | | | | | |  |
| --- | --- | --- | --- | --- | --- | --- | --- | --- | --- | --- | --- | --- | --- | --- | --- | --- | --- |
|  | **1** | **2 ^b^** | **3** | **4 ^b^** | **5** | **6** | **7 ^b^** | **8** | **9 ^b^** | **10** | **11 ^b^** | **12** | **13 ^b^** | **14** | **15 ^b^** | **16** | **Overall rating** |
| Liang ,2024 | Y | N | N | Y | N | N | N | Y | N | Y | Y | Y | N | N | Y | Y | Critically Low |
| Yo Kawaguchi,2021 | Y | N | Y | Y | Y | Y | Y | Y | Y | Y | Y | Y | Y | Y | Y | Y | Moderate |
| Nishimura et al.,2019 | Y | N | PY | Y | Y | Y | Y | Y | Y | N | Y | Y | Y | Y | N | N | Low |
| Fei Chen et al.,2022 | Y | Y | Y | Y | Y | Y | Y | Y | Y | Y | Y | Y | Y | Y | Y | Y | High |
| UzairM. Jogiat et al.,2022 | Y | Y | Y | Y | Y | Y | Y | Y | Y | N | Y | N | Y | Y | Y | N | Moderate |
| Pei-Yu Wang et al.,2020 | Y | Y | Y | Y | Y | Y | Y | Y | Y | Y | Y | Y | Y | Y | Y | Y | High |
| UzairM. Jogiat et al.,2022 | Y | N | N | Y | Y | Y | N | Y | Y | Y | Y | N | Y | Y | Y | Y | Low |
| Chengcong Liu et al.,2025 | Y | N | Y | Y | N | N | Y | Y | Y | Y | Y | N | Y | Y | Y | Y | Moderate |
| Hans-JonasMeyer et al.,2022 | Y | N | N | Y | N | N | Y | Y | Y | Y | Y | N | Y | Y | N | Y | Low |
| Qingyan Kong et al.,2023 | Y | Y | Y | Y | Y | Y | Y | Y | Y | Y | Y | Y | Y | Y | Y | Y | High |
| Hao Zhang et al.,2022 | Y | N | PY | Y | Y | Y | Y | Y | Y | Y | Y | Y | Y | Y | Y | Y | Moderate |
| Jun Jiet et al.,2024 | Y | Y | PY | Y | Y | Y | Y | Y | Y | Y | Y | Y | Y | Y | Y | Y | High |
| Jing Long et al.,2024 | Y | N | N | Y | Y | Y | Y | Y | Y | Y | Y | N | Y | Y | Y | Y | Moderate |
| Chenming Liu et al.,2024 | Y | Y | PY | Y | Y | Y | Y | Y | Y | Y | Y | N | Y | Y | Y | Y | Moderate |
| Zihe Wang et al.,2025 | Y | Y | Y | Y | Y | Y | Y | Y | Y | N | Y | N | Y | Y | Y | N | Moderate |
| Waalboer RB,2023 | Y | N | Y | Y | Y | Y | Y | Y | Y | Y | Y | PY | Y | Y | Y | Y | Moderate |
| Maximilian et al.,2022 | Y | N | N | Y | Y | Y | Y | Y | Y | Y | Y | N | Y | Y | Y | Y | Moderate |
| Clifford Atuiri et al.,2025 | Y | Y | PY | Y | Y | Y | Y | Y | Y | N | Y | N | Y | Y | Y | N | Moderate |
| Claire et al.,2024 | Y | Y | PY | Y | Y | Y | Y | Y | Y | Y | Y | N | Y | Y | Y | Y | Moderate |
| Hans-Jonas Meyer et al.,2022 | Y | N | N | Y | Y | Y | Y | Y | Y | N | Y | N | Y | Y | Y | N | Moderate |
| Michela Roberto et al.,2024 | Y | N | N | Y | Y | Y | Y | Y | N | Y | Y | N | Y | Y | N | Y | Critically Low |
| Mengxing Tian et al.,2025 | Y | N | Y | Y | Y | Y | Y | Y | Y | Y | Y | Y | Y | Y | Y | Y | Moderate |
| Fang Wang et al.,2025 | Y | N | N | Y | Y | Y | Y | Y | Y | Y | Y | Y | Y | Y | Y | Y | Moderate |
| Pedro de PR et al.,2022 | Y | Y | N | Y | Y | Y | Y | Y | Y | Y | Y | N | Y | Y | Y | Y | Moderate |
| Li Yuxuan et al.,2022 | Y | Y | N | Y | Y | Y | PY | Y | Y | Y | Y | N | Y | Y | Y | Y | Moderate |
| Yinghan Zeng et al.,2024 | Y | Y | PY | Y | Y | Y | Y | Y | Y | Y | Y | PY | Y | Y | Y | Y | Moderate |
| Fanyi Qin et al.,2024 | Y | Y | PY | Y | Y | Y | Y | Y | Y | Y | Y | PY | Y | Y | Y | Y | Moderate |
| Hans-Jonas Meyer et al.,2022 | Y | N | PY | Y | N | N | Y | Y | Y | Y | Y | N | Y | Y | N | Y | Low |

Notes:

1. Did the research questions and inclusion criteria for the review include the components of PICO (Population, Intervention, Comparator group, Outcome)? YES/NO. For yes, must have all four.

2. Did the report of the review contain an explicit statement that the review methods were established prior to the conduct of the review and did the report justify any significant deviations from the protocol? YES, PARTIAL YES, NO. For Partial YES: the authors state that they had a written protocol or guide that included ALL the following (review question(s), a search strategy, inclusion/exclusion criteria, a risk of bias assessment). For YES: as for partial yes, plus the protocol should be registered and should also have specified: a meta-analysis/synthesis plan, if appropriate, and a plan for investigating causes of heterogeneity, justification for any deviations from the protocol.

3. Did the review authors explain their selection of the study designs for inclusion in the review? YES/NO. For YES, the review should satisfy one of the following: explanation for including only RCTs, or explanation for including only NRSI, or explanation for including both RCTs and NRSI.

4. Did the review authors use a comprehensive literature search strategy? YES, PARTIAL YES, NO. for PARTIAL YES must have all of the following: searched at least 2 databases (relevant to research question), provided key word and/or search strategy, justified publication restrictions (eg. Language). For YES should also have all of the following: searched the reference lists/biographies of included studies, searched trial/study registries, included/consulted content experts in the field, searched for grey literature where relevant, conducted search within 24 months of completion of the review.

5. Did the review authors perform study selection in duplicate? YES/NO. for YES, either ONE of the following: at least two reviewers independently agreed on selection of eligible studies and achieved consensus on which studies to include OR two reviewers selected a sample of eligible studies and achieved good agreement (at least 80 per cent) with the remainder selected by one reviewer.

6. Did the review authors perform data extraction in duplicate? YES/NO. For YES, either one of the following: at least two reviewers achieved consensus on which data to extract from included studies OR two reviewers extracted data from a sample of eligible studies and achieved good agreement (at least 80 per cent) with the remainder extracted by one reviewer.

7. Did the review authors provide a list of excluded studies to justify the exclusions? YES, PARTIAL YES, NO. FOR partial yes must provide a list of all potentially relevant studies that were read in full text form but excluded from the review. For YES must also have justified the exclusion from the review of each potentially relevant study.

8. Did the review authors describe the included studies in adequate detail? YES, PARTIAL YES, NO. For PARTIAL YES, must describe all of the following: populations, interventions, comparators, outcomes, research designs. For YES should also have all of the following: described populations in detail, described intervention and comparator in detail (including doses where relevant), described study setting, timeframe or follow-up.

9. Did the review authors use a satisfactory technique for assessing the risk of bias (RoB) in individual studies that were included in the review? For RCTs: YES, PARTIAL YES, NO, INCLUDES ONLY NRSI. For PARTIAL YES must have assessed RoB from unconcealed allocation and lack of blinding of patients and assessors when assessing outcomes (unnecessary for objective outcomes such as all cause mortality); for YES must also have assessed RoB from allocation sequence that was not truly random and selection of the reported result from among multiple measurements or analyses of a specified outcome. For NRSI (Non Randomized Studies of Intervention): YES, PARTIAL YES, NO, INCLUDES ONLY RCTs. For PARTIAL YES must have assessed RoB from confounding and from selection bias. For YES, must also have assessed methods used to ascertain exposures and outcomes, and selection of the reported results from among multiple measurements or analyses of a specified outcome.

10. Did the review authors report on the sources of funding for the studies included in the review? YES/NO. For YES: must have reported on the sources of funding for individual studies included in the review. Note: reporting that the reviewers looked for this information but it was not reported by study authors also qualifies

11. If meta-analysis was performed, did the review authors use appropriate methods for statistical combination of results? For RCTs: YES, NO, NO META-ANALYSIS. For YES: the authors justified combining the data in a meta-analysis and they used an appropriate weighted technique to combine study results and adjusted for heterogeneity if present and investigated the causes of heterogeneity. For NRSI: YES, NO, NO META-ANALYSIS CONDUCTED. For YES: the authors justified combining the data in a meta-analysis and they used an appropriate weighted technique to combine study results, adjusting for heterogeneity if present, and they statistically combined effects estimates from NRSI that were adjusted for confounding, rather than combining raw data, or justified combining raw data when adjusted effect estimates were not available, and they reported separate summary estimates for RCTs and NRSI separately when both were included in the review.

12. If meta-analysis was performed, did the review authors assess the potential impact of RoB in individual studies on the results of the meta-analysis or other evidence synthesis? YES, NO, NO META-ANALYSIS INCLUDED. For YES: included only low risk of bias RCTs or, if the pooled estimate was based on RCTs and/or NRSI at variable RoB, the authors performed analysis ton investigate possible impact of RoB on summary estimates of effect.

13. Did the review authors account for RoB in individual studies when interpreting/discussing the results of the review? YES/NO. for YES: included only low risk of bias RCTs or, if RCTs with moderate or high RoB, or NRSI were included, the review provided a discussion of the key impact of RoB on the results

14. Did the review authors provide a satisfactory explanation for, and discussion of, any heterogeneity observed in the results of the review? YES/NO. For Yes: there was no significant heterogeneity in the results OR if heterogeneity was present the authors performed an investigation of sources of any heterogeneity in the results and discussed the impact of this on the results of the review

15. If they performed quantitative synthesis did the review authors carry out an adequate investigation of publication bias (small study bias) and discuss its likely impact on the results of the review? YES, NO, NO META-ANALYSIS CONDUCTED. For YES: performed graphical statistical tests for publication bias and discussed the likelihood and magnitude of impact of publication bias

16. Did the review authors report any potential sources of conflict of interest, including any funding they received for conducting the review? YES/NO. For Yes: the authors reported no competing interests OR the authors described their funding sources and how they managed potential conflicts of interest.

d Rating overall confidence in the results of the review:

HIGH: no on one non-critical weakness: the systematic review provides an accurate and comprehensive summary of the results of the available studies that address the question of interest

MODERATE: more than one non critical weakness (multiple non-critical weaknesses may diminish confidence in the review and it may be appropriate to move the overall appraisal down from moderate to low confidence): the systematic review has more than one weakness but no critical flaws. It may provide an accurate summary of the results of the available studies that were included in the review

LOW: one critical flaw with or without non-critical weaknesses: the review has a critical flaw and may not provide an accurate and comprehensive summary of the available studies that address the question of interest

CRITICALLY LOW: more than one critical flaw with or without non-critical weaknesses: the review has more than one critical flaw and should not be relied on to provide an accurate and comprehensive summary of the available studies.


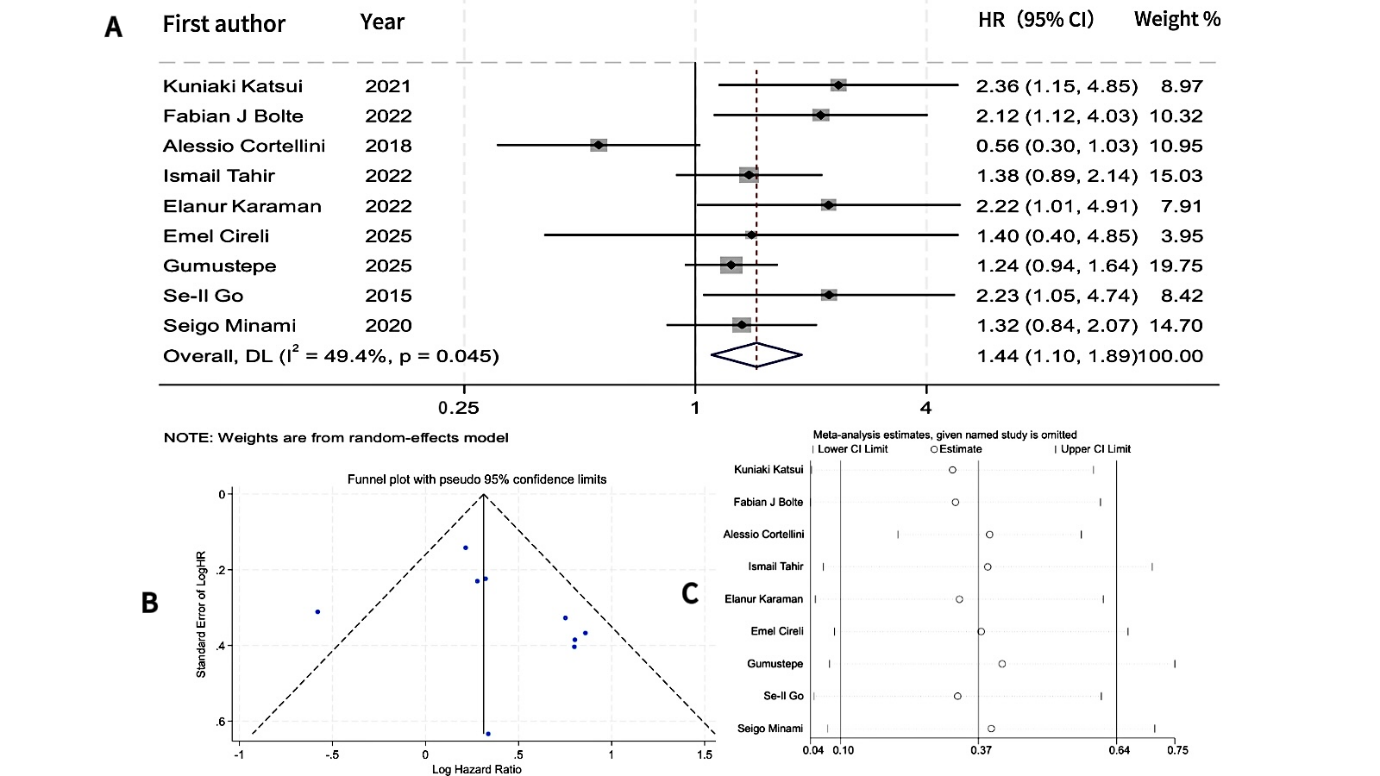


**Supplement Figure S1: Overall Survival After First-Line Therapy in Lung Cancer.**

**A**. Forest plot. **B**. Funnel plot for publication bias. **C**. Leave-one-out sensitivity analysis.

HR, hazard ratio; CI, confidence interval; non-RCT, non-randomized controlled trial.

**
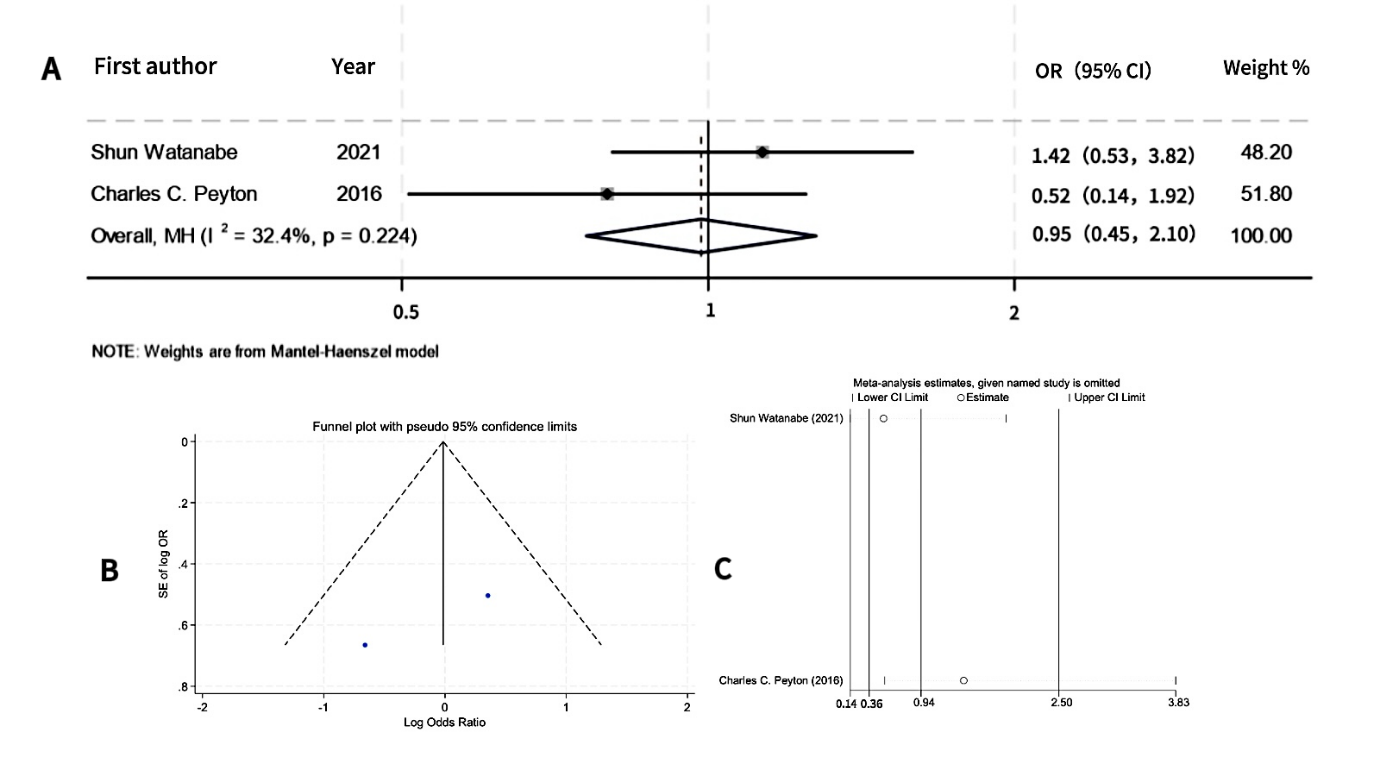
**

**Supplement Figure S2: Progression Free Survival After First-Line Therapy in Lung Cancer.**

**A**. Forest plot. **B**. Funnel plot for publication bias. **C**. Leave-one-out sensitivity analysis.

HR, hazard ratio; CI, confidence interval;


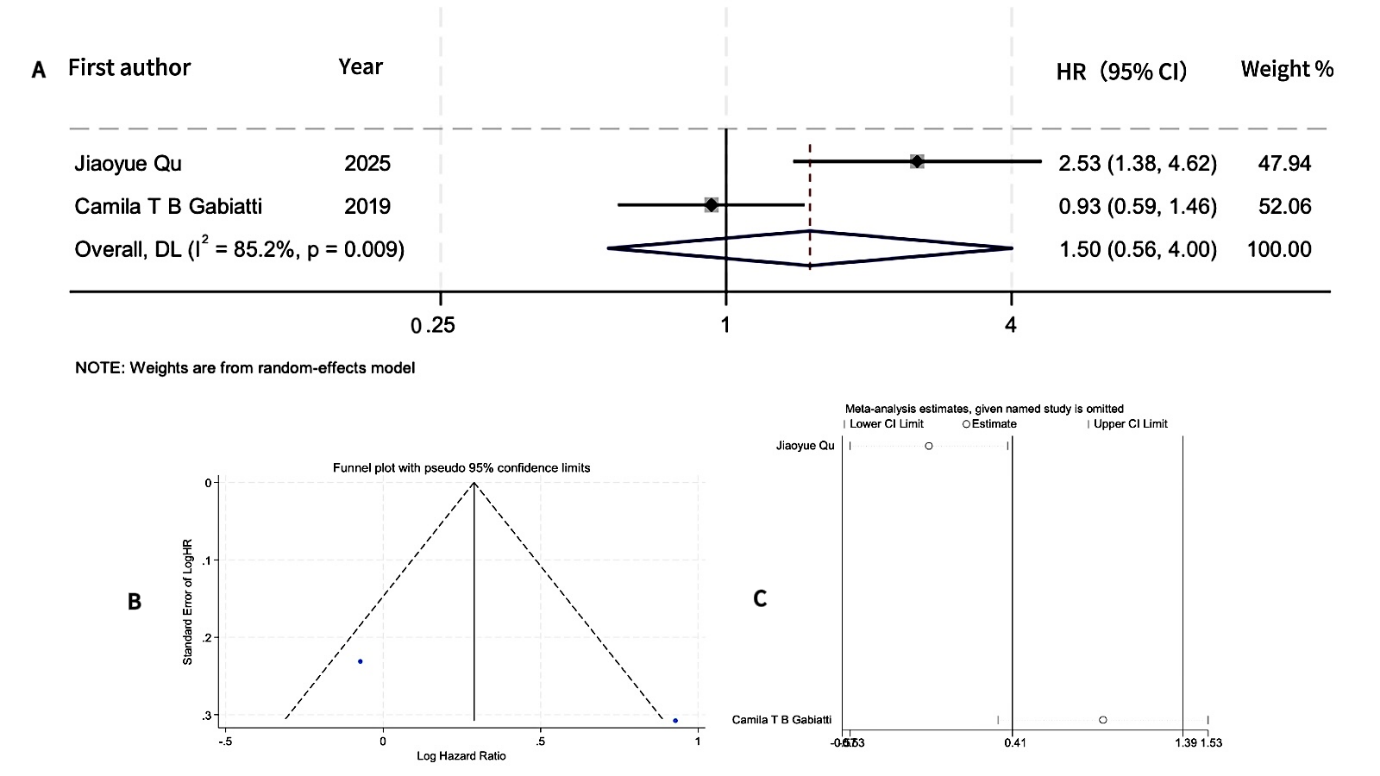


**Supplement Figure S3: Progression Free Survival After First-Line Therapy in Esophageal Cancer.**

**A**. Forest plot. **B**. Funnel plot for publication bias. **C**. Leave-one-out sensitivity analysis.

HR, hazard ratio; CI, confidence interval;


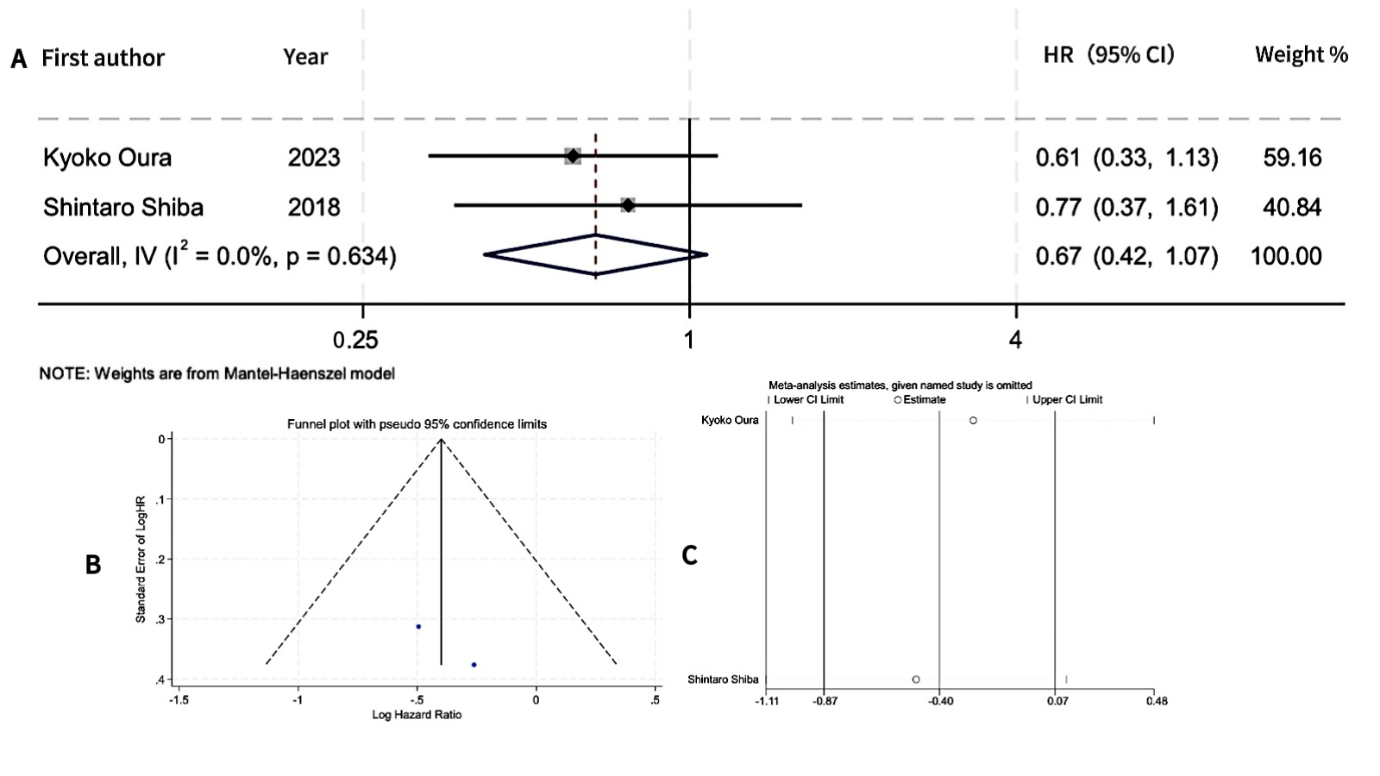


**Supplement Figure S4: Progression Free Survival After First-Line Therapy in Hepatocellular Carcinoma.**

**A**. Forest plot. **B**. Funnel plot for publication bias. **C**. Leave-one-out sensitivity analysis.

HR, hazard ratio; CI, confidence interval;


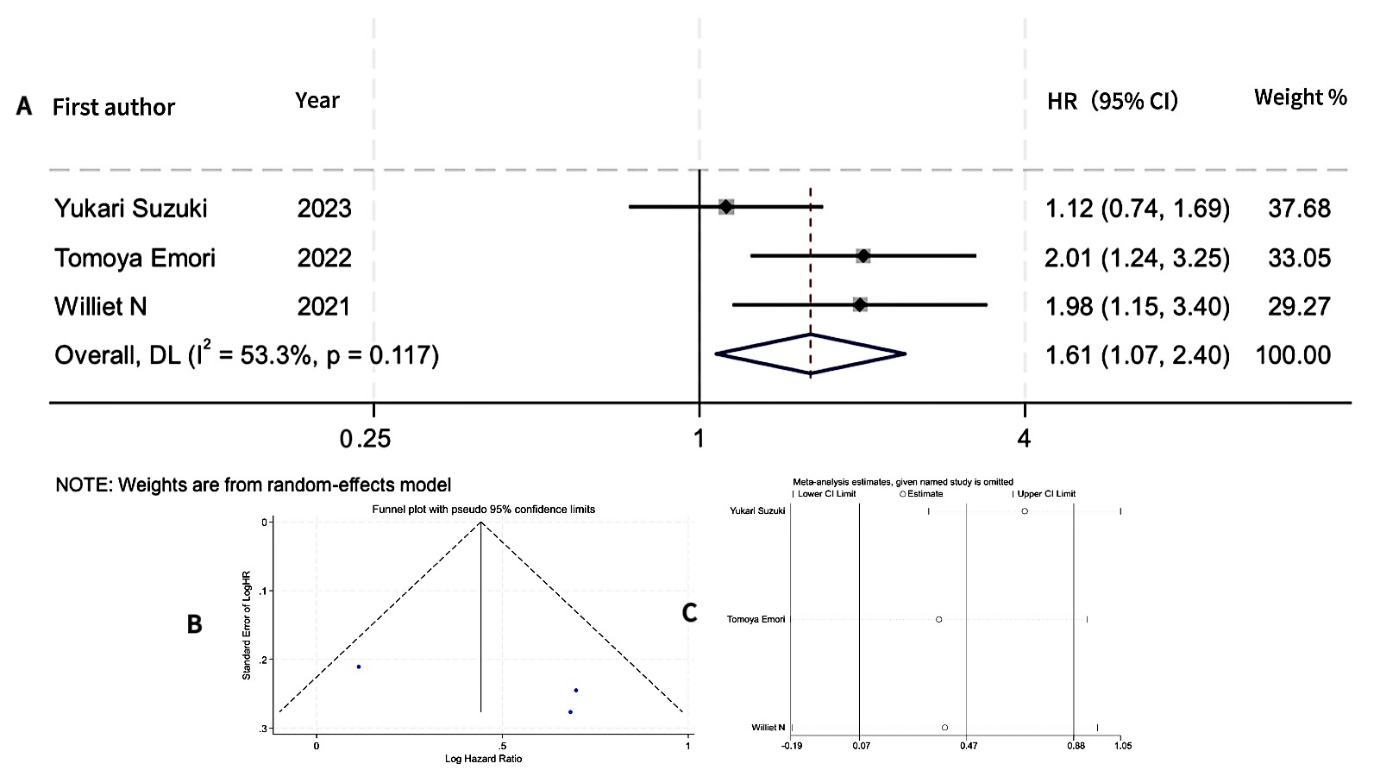


**Supplement Figure S5: Progression Free Survival After First-Line** **Therapy in Pancreatic Carcinoma.**

**A**. Forest plot. **B**. Funnel plot for publication bias. **C**. Leave-one-out sensitivity analysis.

HR, hazard ratio; CI, confidence interval;


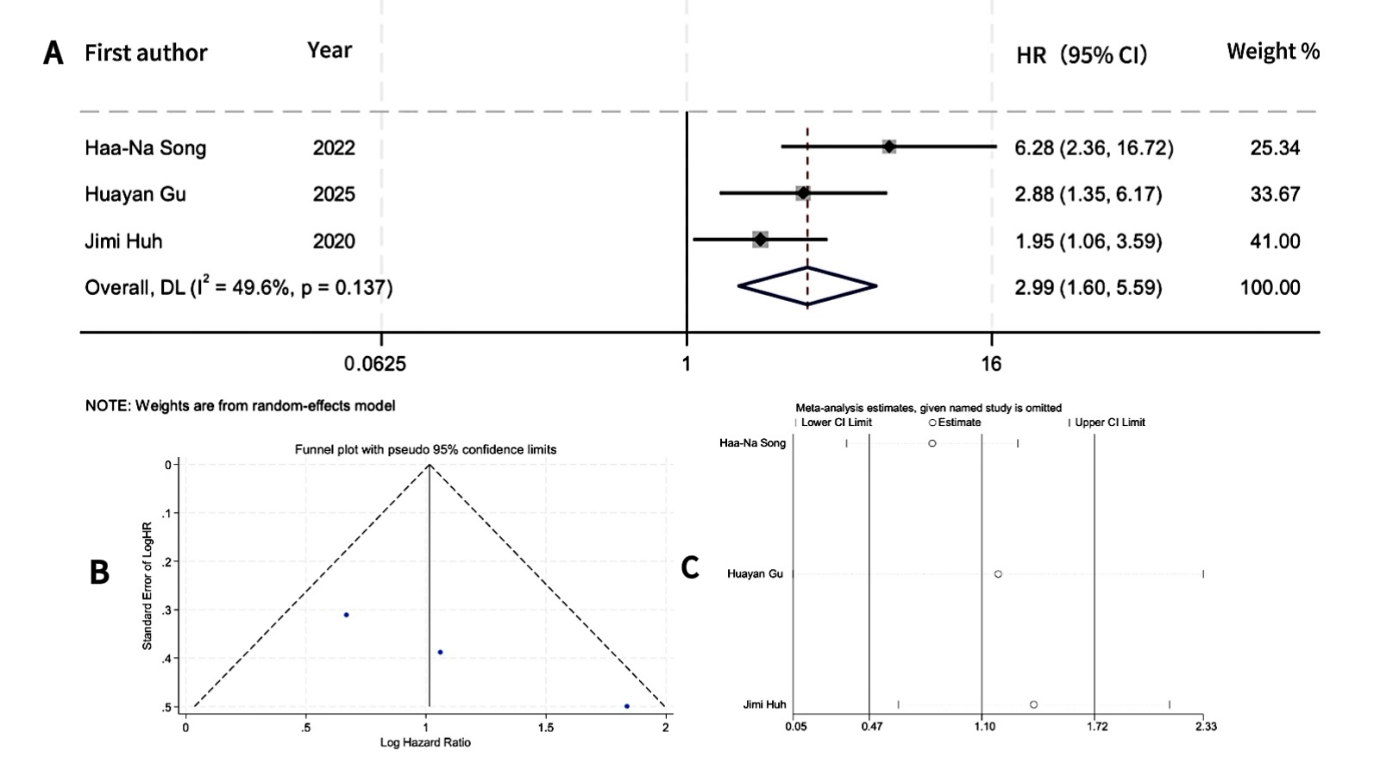


**Supplement Figure S6: Overall Survival After Surgery in Breast Cancer.**

**A**. Forest plot. **B**. Funnel plot for publication bias. **C**. Leave-one-out sensitivity analysis.

HR, hazard ratio; CI, confidence interval;


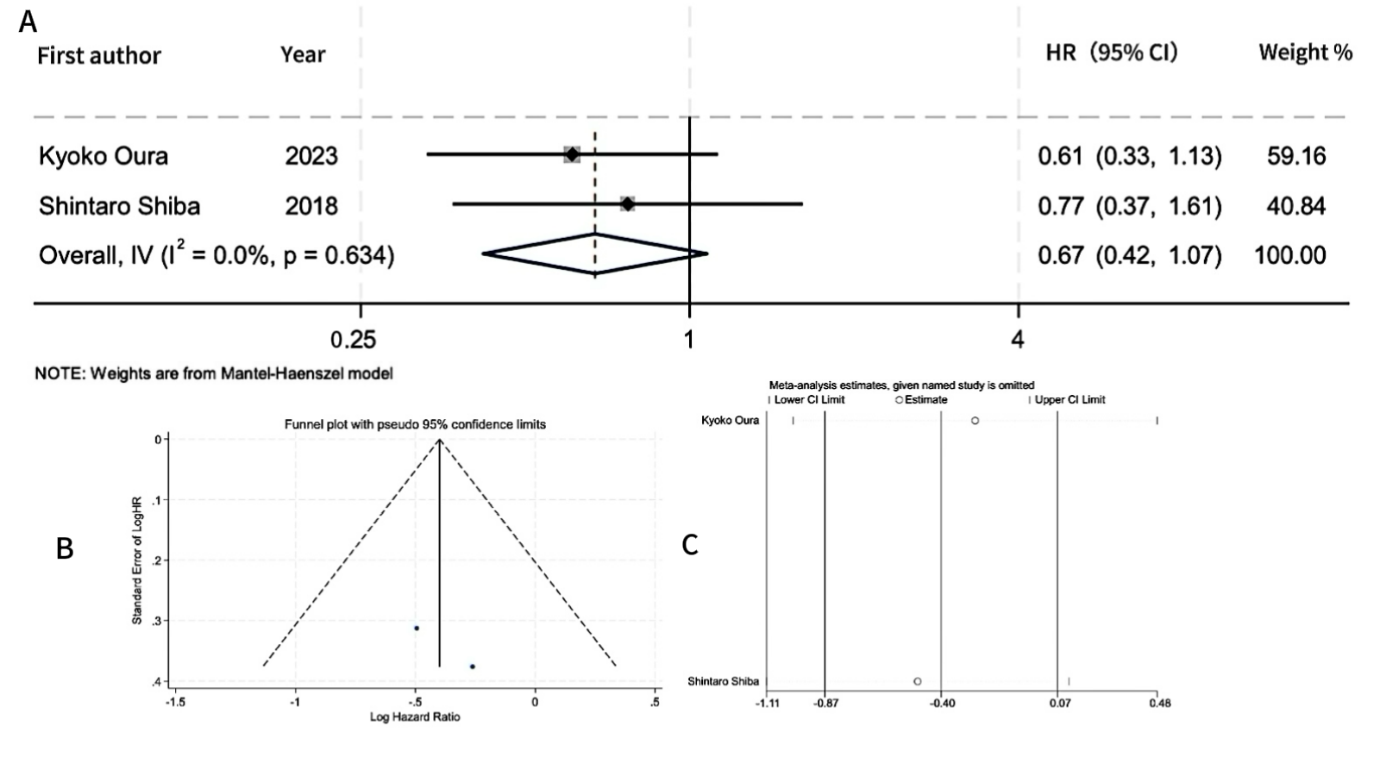


**Supplement Figure S7: Disease Free Survival After Surgery in Breast Cancer.**

**A**. Forest plot. **B**. Funnel plot for publication bias. **C**. Leave-one-out sensitivity analysis.

HR, hazard ratio; CI, confidence interval;


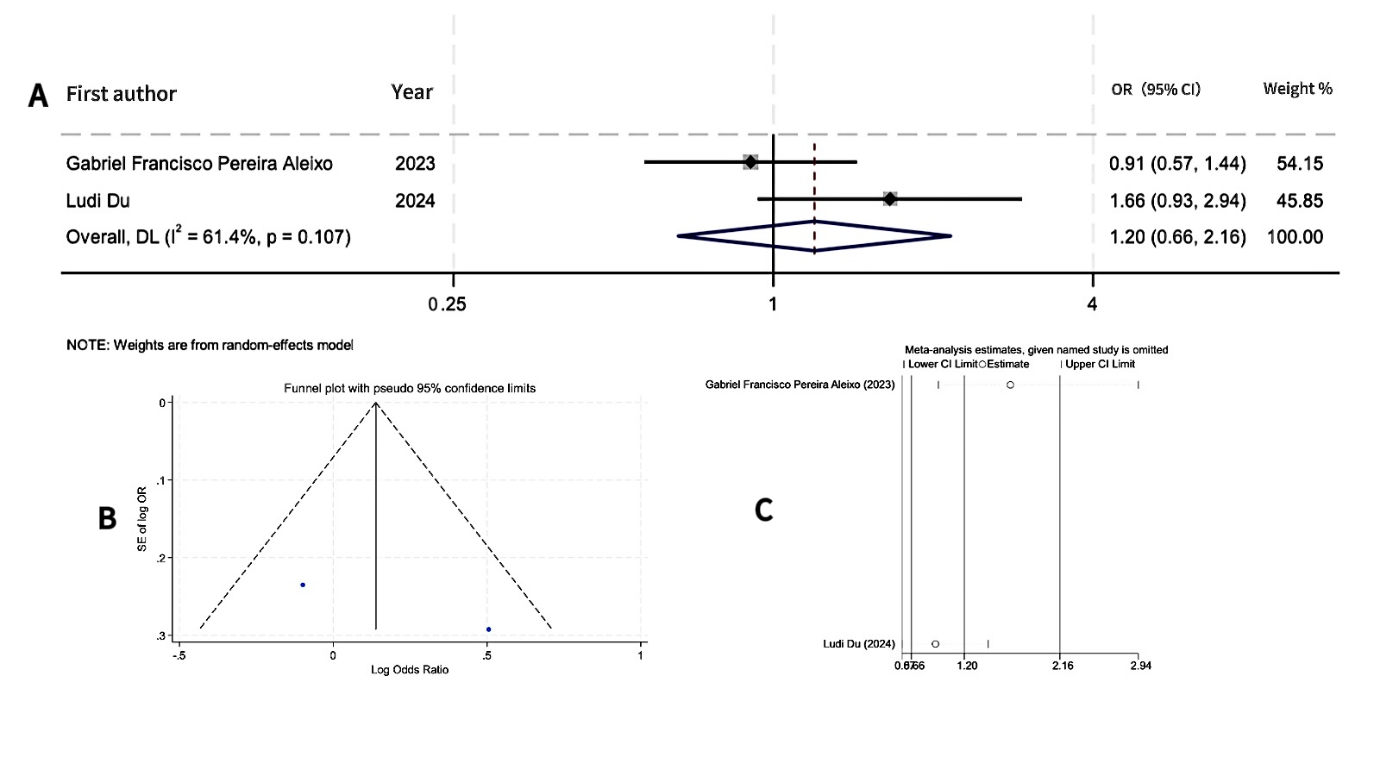


**Supplement Figure S8: Postoperative Complications in Breast Cancer.**

**A**. Forest plot. **B**. Funnel plot for publication bias. **C**. Leave-one-out sensitivity analysis.

HR, hazard ratio; CI, confidence interval;


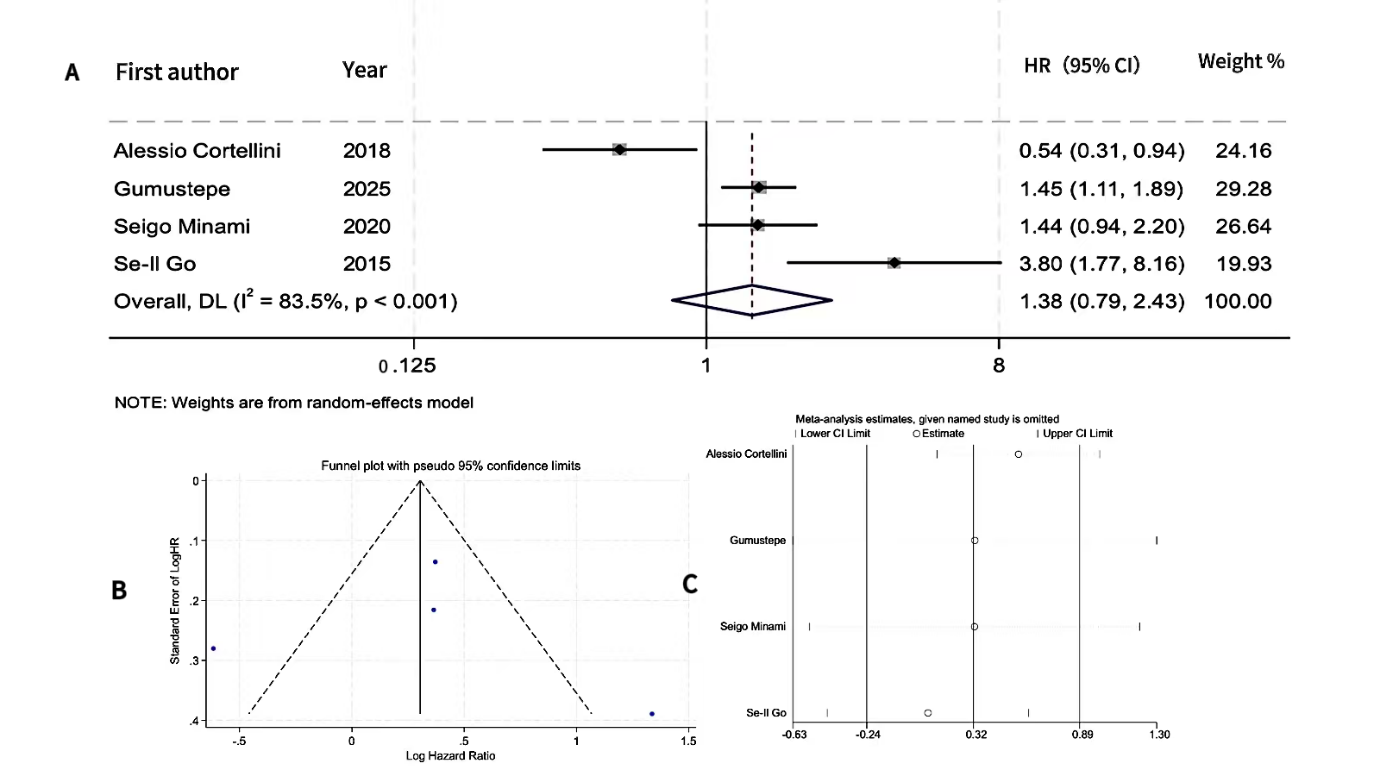


**Supplement Figure S9: Postoperative Complications in Renal Cell Carcinoma.**

**A**. Forest plot. **B**. Funnel plot for publication bias. **C**. Leave-one-out sensitivity analysis.

HR, hazard ratio; CI, confidence interval;


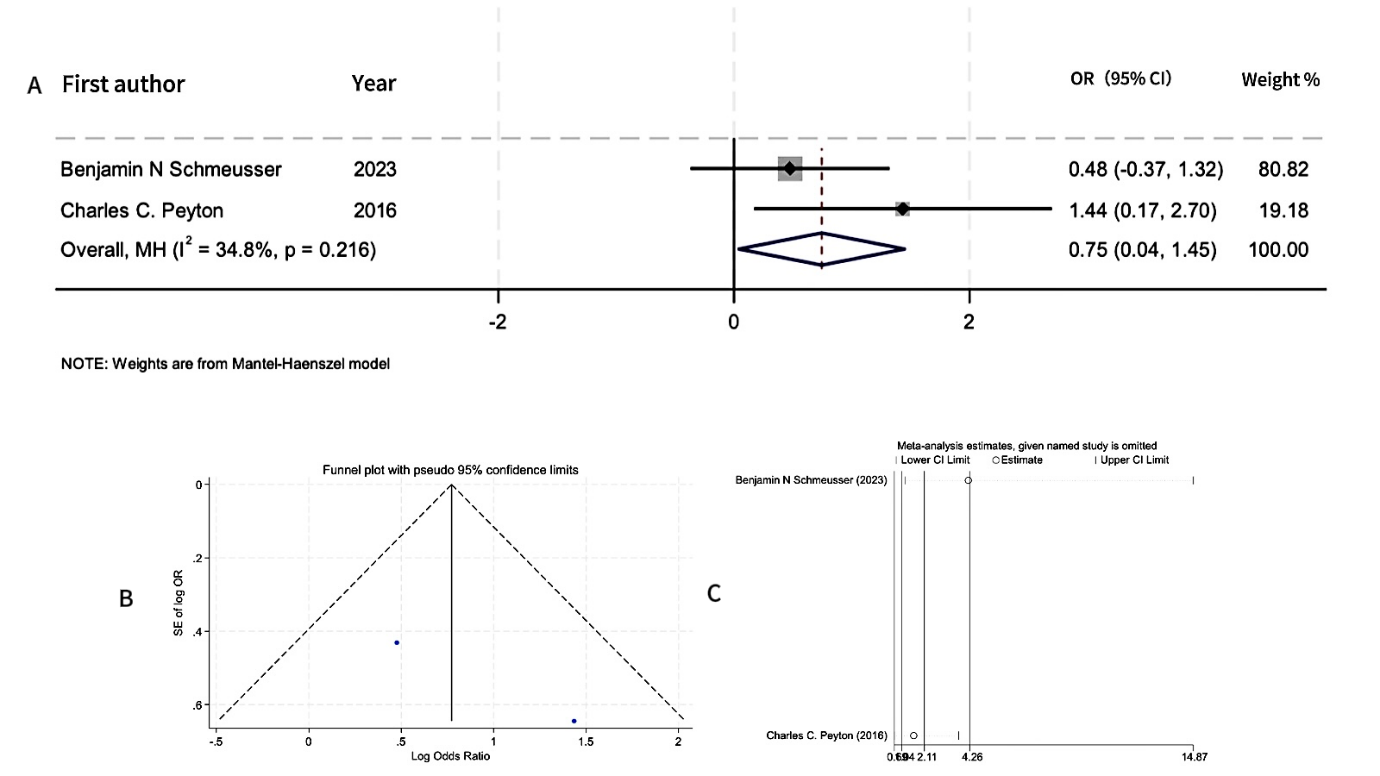


**Supplement Figure S10: Postoperative Major Complications in Renal Cell Carcinoma.**

**A**. Forest plot. **B**. Funnel plot for publication bias. **C**. Leave-one-out sensitivity analysis.

HR, hazard ratio; CI, confidence interval;
